# The Relationship between Addictive Eating and Dietary Intake: A Systematic Review

**DOI:** 10.3390/nu14010164

**Published:** 2021-12-30

**Authors:** Kirrilly M. Pursey, Janelle Skinner, Mark Leary, Tracy Burrows

**Affiliations:** 1 Nutrition and Dietetics, School of Health Sciences, College of Health, Medicine and Wellbeing, The University of Newcastle, Callaghan, NSW 2308, Australia; janelle.skinner@newcastle.edu.au (J.S.); mark.leary@newcastle.edu.au (M.L.); tracy.burrows@newcastle.edu.au (T.B.); 2 Priority Research Centre for Physical Activity and Nutrition, The University of Newcastle, Callaghan, NSW 2308, Australia

**Keywords:** addictive eating, food addiction, dietary intake

## Abstract

(1) Background: Research suggests that certain foods may have addictive effects; however, no reviews have systematically appraised studies in this area. The aims of this review were to determine the nutrients, foods and dietary patterns associated with addictive eating. (2) Methods: Published studies up to November 2020 were identified through searches of 6 electronic databases. Eligible studies included those in in children and adults that reported dietary intakes of individuals with ‘food addiction’. (3) Results: Fifteen studies (*n* = 12 in adults and *n* = 3 in children/adolescents with Yale Food Addiction Scale defined ‘food addiction’) were included. Foods commonly associated with addictive eating were those high in a combination of fat and refined carbohydrates. Generally, intakes of energy, carbohydrates and fats were significantly higher in individuals with addictive eating compared to those without. (4) Conclusions: Due to the heterogeneity in study methodologies and outcomes across included studies, it is difficult to conclude if any specific foods, nutrients or dietary patterns facilitate an addictive process. Further research is needed to elucidate potential associations. However, present addictive eating treatment approaches could incorporate individualised dietary advice targeting foods high in fat and refined carbohydrates.

## 1. Introduction

A growing body of evidence suggests that there are behavioral, neurobiological, and genetic overlaps between the consumption of certain foods and addiction-related disorders [1,2,3,4]. The term “food addiction” has been used to describe certain eating patterns that resemble addictive-related disorders and has frequently been operationalized using the DSM criteria for substance use disorders [1]. Existing FA research has predominantly focused on the prevalence in different population groups including adults and adolescents using self-report tools [5,6,7]. However, there is increasing interest in the individual foods and dietary profiles associated with addictive eating [8]. It has been suggested that certain foods or ingredients may have the potential to facilitate an addictive-like response in susceptible individuals. Ongoing debate centres around if the construct of FA indeed resembles a substance-related disorder that is facilitated by certain ingredients or components in foods, or whether it is better conceptualized as a behavioural addiction, whereby it is the compulsive overconsumption of a variety of foods irrespective of nutritional composition (for further discussion see [9,10,11,12,13]. As it has been posited that addictive-like eating may have the potential to facilitate overeating and weight gain in certain individuals [2], it is important to evaluate whether certain components within foods may trigger an addictive-like response to better inform future FA treatments. 

The foods and nutrients most often cited as being addictive both within the scientific community and general public include refined carbohydrates (i.e., sugar), salt and/or fat, or combinations of these [8,14,15]. These foods are thought to exceed the rewarding properties of traditional foods, such as vegetables, fruits, and nuts due to the high potency of refined ingredients [16]. An extensive body of evidence derived from animal studies supports the viewpoint that food and beverages considered to have an addiction potential are those that contain large quantities of sugar. This is due to its activation of opioid receptors and ability to foster tolerance, withdrawal and cross-sensitisation in a similar way to addictive drugs [17]. High fat foods have also been shown to induce binge eating in rats when given intermittently [18]. However, this has not yet been replicated consistently in human studies. This may be due the limited generalizability of animal studies to the human context as they do not account for the environmental and social aspects of eating. A narrative review of the evidence suggested that rather than individual macronutrients alone (i.e., sugar and fat), it is highly processed, hyper-palatable foods with combinations of fat, sugar and salt that may have addictive effects [8]. Further, levels of processing and glycaemic index were important factors to consider when evaluating the addictive potential of foods [8]. However, no systematic reviews have yet confirmed the findings of this narrative review. 

Though researchers and clinicians often associate addictive eating with ‘ultra-processed foods’ or highly palatable foods, these terms are often used without a definition. With preclinical and clinical evidence now supporting a potential link between reward driven eating and processed foods [19], more stringent use of terms should be considered by researchers in the field to avoid confusion or misclassification of foods. One such international classification system, known as NOVA [20], has grown in influence over the last few years [21] due to the strong associations found between processed foods and adverse health outcomes [22]. However, the NOVA system centres primarily around the level of processing, rather than the classification of foods in terms of ingredients or nutrients, which may or may not fully assist in clarifying the debate as to whether FA is better described as a substance-related or behavioural disorder. It is also important to consider the dietary assessment methods used to assess dietary intakes in individuals with FA. As there are many considerations when choosing a dietary assessment tool, such as whether short- or longer term/habitual intakes are assessed and validity of the tool for the population being sampled [23], it is important to evaluate this and identify potential research gaps within the FA field. 

Thus, the evidence for the types of foods associated with addictive eating, and the validity of dietary assessment measures used to ascertain these, remains unclear. It is timely to synthesise existing evidence regarding dietary assessment methods used, intakes associated with FA to improve the rigour and understanding in the field, and potentially inform future nutrition-related treatment approaches. It is also of importance to assess dietary intakes associated with FA across the differing life stages due to differences in dietary intakes across ages. This systematic review aimed to synthesise published studies assessing nutrients, foods and dietary patterns associated with addictive eating in children and adults.

## 2. Materials and Methods

### 2.1. Search Strategy

A systematic search strategy was conducted from the time of database inception to November 2020. Six databases were searched including Cochrane Database, EMBASE (Excerpta Medica Database), MEDLINE (Medical Literature Analysis and Retrieval System Online), PsycINFO (Psychological Information Database), Scopus, and Web of Science. Two sets of terms were used for the search strategy, (1) terms relating to addictive eating; and (2) terms relating to dietary intake. Searches were limited to humans and publications in the English language. An example of the search strategy is available in Supplementary material (Table S1). The review methodology was registered with OSF (Open Science Framework) Registries (https://osf.io/uvy84 (accessed on 10 December 2020)) and conducted in accordance with the Preferred Reporting Items for Systematic Reviews and Meta-Analyses (PRISMA) Guidelines [24]. 

### 2.2. Study Criteria

Studies in males and females of all ages were included in this review if they specifically assessed addictive eating using a valid assessment method or tool (e.g., self-report tools such as the Yale Food Addiction Scale [25] or Addiction-like Eating Behaviour Scale [26]) in combination with dietary intake (i.e., reporting energy, nutrient or food intake, dietary patterns or diet quality), see Table 1 for PICOS criteria. Randomised controlled trials (RCT), non-randomised or quasi-randomised controlled trials, cohort studies and pre-post studies that used a non-food addicted group as a comparator were included, as well as studies that used no comparator. Narrative reviews, theses, conference proceedings, commentaries, letters to the editor, and studies with inadequate information regarding the methodological details of the study were excluded from the review. Studies that investigated participants with a self-identified food addiction without the use of a tool (e.g., self-described ‘chocoholics’) were excluded from the review.

### 2.3. Study Selection

After the removal of duplicates, identified studies were imported into COVIDence web-based software (www.COVIDence.org (accessed on 10 December 2020)). Titles and abstracts were screened by two independent reviewers (KP, ML, RN, or JS). Full text articles were subsequently retrieved and screened by two reviewers (KP, ML, RN, or JS) for inclusion in the review. In any cases of uncertainty about a study’s inclusion in the review, a third reviewer was consulted until consensus was reached (KP, JS or TB). 

### 2.4. Data Extraction and Synthesis

Data extraction was conducted using a standardised table developed for this review, and pilot tested on three randomly selected included studies, with no modifications required. Data extraction included study design, sample characteristics, intervention details, outcomes relating to food addiction and dietary intake, follow-up duration and study limitations. One review author extracted the data from included studies, and a second author independently checked the extracted data (KP, JS or TB). Studies were synthesised in a narrative summary. Studies were grouped according to age (adolescents < 18 years of age versus adults 18 years and older) and participant sex (male versus female) for subgroup analysis. Due to an insufficient number of studies with similar dietary outcomes and comparable dietary assessment methodology, a meta-analysis of primary outcomes measures was not able to be conducted. 

### 2.5. Quality of Evidence

Quality of retrieved studies was assessed by two independent reviewers (JS and TB) using the Academy of Nutrition and Dietetics Quality Criteria Checklist for Primary Research [27], a standardised 10-item tool that can be applied to a broad range of study designs. This checklist includes ten criteria which relate to the presence or absence of threats to the validity of research including clarity of the research question; subject selection; comparability of study groups; handling of withdrawals; blinding; descriptions of the intervention; validity of outcome measures; appropriateness of statistical methods and data synthesis; conclusions drawn; and likelihood of funding bias. Each item was classified as present “Yes” (high risk of bias), “No” (low risk of bias), absent or “Unclear” for each included study. The overall study quality was then rated as “Positive” (i.e., low risk of bias) if criteria 2, 3, 6, 7, and one other were “Yes”, “Neutral” if criteria 2, 3, 6, and/or 7 are “No”, “Unclear”, or “Negative” (i.e., high risk of bias) if six or more criteria were “No”. Discrepancies were resolved through discussion amongst the independent reviewers and no studies were excluded based on quality ratings.

## 3. Results

### 3.1. Description of Included Studies

**Figure 1:**
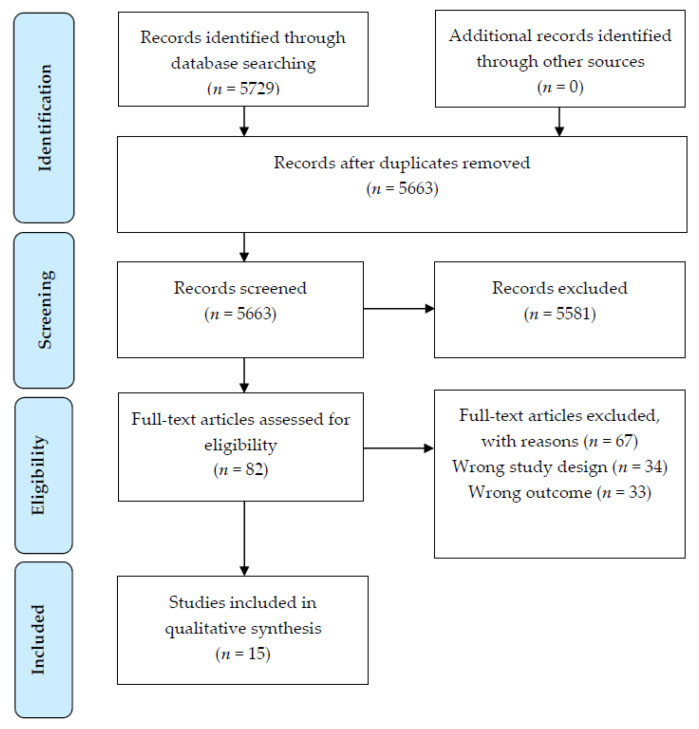
PRISMA [19] flow diagram of article identification retrieval and inclusion for the systematic review.

In total, 5729 articles were identified during the search. Following assessment against the inclusion criteria, 15 articles were included in this review (Figure 1). The majority of included studies used a cross-sectional design (*n* = 12 studies [28,29,30,31,32,33,34,35,36,37,38,39]); with the remaining being a RCT (*n* = 1 [40]) and two prospective cohort studies (*n* = 1 study, with 3, 6 and 12-month follow-up of dietary and FA outcomes [41]; and *n* = 1 study, with a cross-sectional analysis of data collected [42]) In descending order, studies were carried out in Turkey (*n* = 4), Australia (*n* = 3), Canada (*n* = 2), USA (*n* = 2), Brazil (*n* = 1), Greece (*n* = 1), Iran (*n* = 1), and Israel (*n* = 1), Table 2.

### 3.2. Quality of Included Studies

The quality assessment appraisals of included studies deemed 13 studies as having a positive rating, and two as having a neutral rating (See Supplementary material, Table S2). Overall, the studies rated as neutral did not provide sufficient details of dietary assessment tools used to determine if measurements were based on standard, valid, and reliable data collection instruments. Additionally, characteristics of withdrawals (i.e., response rate for cross-sectional studies, *n* = 2); study limitations or sources of funding were not described or disclosed. 

### 3.3. Participant Characteristics

A total of 128,441 participants (*n* = 1395 male and 127,046 female) were included across the studies, and study sample sizes ranged from 18 to 123,688 (median, 181). Eleven of the 15 studies included both male and female participants [28,29,30,31,32,33,35,36,37,38,39], and four studies included female participants exclusively [34,40,41,42]. Twelve of the included studies were carried out exclusively in adults ranging in age from 18 to 91 years (mean, 36.2 ± 7.3; *n* = 11 studies reported mean age), and three studies exclusively in children/adolescents ranging in age from 9 to 18 years (mean, 13.0 ± 2.3) [30,32,38]. Six studies included participants recruited from the general community [28,29,35,36,37,40]; three studies recruited participants from schools/universities [30,31,39], with one sample of university students including undergraduate students from the Nutrition department only [31]. Three studies included participants seeking weight loss (*n* = 2 studies in children/adolescents [32,38], and *n* = 1 study in adults [34]); one included adults undergoing bariatric surgery [41]; and one included adults with schizophrenia [33]. For all studies, with the exception of one [39], participants’ weight status was reported, with six studies including participants classified as within the overweight or obese body mass index (BMI) category exclusively [30,32,34,35,38,41]. Across the included studies, mean BMI was 30.6 kg/m^2^ (i.e., within the obese BMI category; range 17.0 to 78.3) and mean BMI z-score for adolescent studies was 2.3 (*n* = 2 studies). The mean proportion of participants within the underweight, healthy weight, overweight and obese BMI categories as reported in studies was 2.1%, 34.6%, 32.2%, 31.1%, respectively.

### 3.4. Addictive Eating Assessment and Outcomes

All included studies used the Yale Food Addiction Scale (YFAS) to assess addictive eating, which provides two scoring options: (1) a ‘symptom score’ reflecting the number of addiction-like criteria endorsed, and (2) a dichotomous food addiction ‘diagnosis’. Nine studies used the original version of the YFAS, two used the YFAS 2.0, two used the modified version of the YFAS (mYFAS) and two used the YFAS for children (YFAS-C). The prevalence of FA diagnosis, across the 15 studies, ranged from 2.5–71.0%. The highest prevalence rates were reported in samples of children/adolescents attending an outpatient clinic for obesity (*n* = 100, mean prevalence 71.0%) [32] and in adults diagnosed with schizophrenia (*n* = 104, mean prevalence 60.6%) [33]. Nine studies reported prevalence by sex, with females having a higher prevalence than males (mean = 18.9%, range: 6.7–42.0% versus mean = 11.2%, range 1.0–29.0%), although this difference was only statistically significant in three of the studies [29,36,37]. Seven studies [28,29,34,36,37,38,40] reported higher FA symptom scores and/or FA diagnosis were associated with higher BMI, whereas five studies [30,31,32,33,41] reported no significant association.

### 3.5. Dietary Intake Assessment and Outcomes

Dietary intake was assessed prospectively in three studies, and retrospectively in 12 studies. Collection of dietary data ranged from short term (24 h to 1 week, *n* = 5 studies) to longer term (1 month to 12 months, *n* = 5 studies) collection periods. Five studies did not specify reporting period. Prospective dietary assessment methods included 3-day food diary or record, but it was not specified whether weekend days and/or weekdays were assessed or if an average of the three days was used to evaluate dietary intakes. The majority of studies used food frequency questionnaires (FFQ; *n* = 10) to retrospectively assess dietary intake. Of these studies, eight cited validation studies in general populations for the FFQ used. No study cited validation of a FFQ tool used in a FA population. One study used standardised questions taken from a national health survey. The remaining study used a dietitian administered 24 h recall with misreporters excluded from analysis using Goldberg cut-offs. With the exception of two studies, misreporting was not assessed in any of the included studies. Of the 13 studies assessing energy and/or nutrient intakes, all but one study specified the food composition database and/or analytical software used to estimate the energy and nutrient content of foods consumed.

The most common dietary outcome measures were energy intake (kJ/day and/or kcal/day; *n* = 11 studies) and macronutrient intakes (g/day, % energy/day or g/kg body weight of protein, fat and/or carbohydrates; *n* = 11 studies). Other dietary outcomes included individual dietary fats (g/day or mg/day of saturated fat, monounsaturated fat, polyunsaturated fat, trans fat, omega 3, omega 6 and/or cholesterol; *n* = 8 studies), dietary fibre (*n* = 7 studies), sugar (total sugars, added sugars, and/or fructose; *n* = 5 studies), and micronutrients (mg/day, µg/day or IU/day; *n* = 6 studies). Seven studies assessed intakes of specific food items [*n* = 1 study, ultra-processed foods and unprocessed/minimally processed foods as defined by the NOVA classification system; *n* = 1 study, positively reinforcing foods identified from published research; *n* = 1 study, select energy-dense foods/beverages (classification system for foods chosen not specified)] or food groups/categories [*n* = 3 studies, core and non-core foods as defined by the Australian Dietary Guidelines; *n* = 1 study, food groups (classification system for food groups not specified), with two studies also reporting diet quality scores. One study reported foods that participants self-reported as the ‘most addictive’.

### 3.6. Relationships and Associations between Addictive Eating and Dietary Outcomes

All the included studies reported the mean difference in dietary outcome variables (i.e., daily intakes of energy, macronutrients, micronutrients, dietary scores and/or specific foods/food groups) between individuals with and without a YFAS defined FA ‘diagnosis’ (See Supplementary material: Table S3. Dietary Outcomes of Included Studies). Four studies reported the association (odds ratios) between FA diagnosis and intakes of specific foods/food groups, and one study reported the association (odds ratios) between FA diagnosis and energy, and nutrient intakes. Three studies reported associations (Pearson’s or Spearman’s rank-order correlations; or Cohen’s d effect size) between YFAS FA symptom scores and energy, and nutrient intakes.

#### 3.6.1. Energy Intake

Across the 11 studies examining energy intake, six studies reported significantly higher daily intakes in adults [28,33,34,35,39] and adolescents [38] with FA compared to NFA (mean difference ranged from 306 to 689 kcal/day). In two studies, this finding was statistically significant in females only [28,29]. Two studies reported significant positive correlations between YFAS symptom scores and energy intake in adults [39] and adolescents [38] (rs = 0.23 and r = 0.23, respectively). No significant differences were reported in one study in children [30] and three studies in adults [31,37,41]; with one study in adults [37] also reporting that energy intake was not significantly associated with higher odds of FA. Significance was not tested in one study [42].

#### 3.6.2. Macronutrient Intakes

A range of macronutrients were assessed across 11 studies. As described below, while some significant differences and associations with FA were reported across studies, findings were not consistent. *Protein*: Ten studies assessed protein intake. Four studies [28,34,36], including one study in children [30], reported significantly higher intakes of protein (g/day or % energy/day) in FA compared to NFA. In one study [28] this finding was significant in females only, and in another study [36] when intake (% energy/day) was expressed as g/kg of body weight the difference was no longer significant. Six studies [31,33,35,37,39,41] reported no significant difference in daily protein intakes (g/day or % energy/day) between those with and without FA, with one study in adults [37] also reporting that protein intake was not significantly associated with higher odds of FA. Two studies [37,39] reported YFAS symptom scores were not significantly associated with protein intake. *Carbohydrates*: Eleven studies assessed carbohydrate intake. In the two studies examining associations, higher YFAS scores were associated with higher intakes of carbohydrates in both male and female adults (r = 0.25 and r = 0.17, respectively) [39], and adolescents (rs = 0.20) [38]. Seven studies [33,34,35,39,41], including one study in children [30] and one study in adolescents [38], reported significantly higher intakes of carbohydrates (g/day, *n* = 6 studies; or g/kg BW, *n* = 1 study) in FA compared to NFA. In one study this finding was significant in females only [39], and in a sample of individuals undergoing bariatric surgery [41], the difference was only significant at the 12 months post-surgery follow-up, but not at the pre-surgery baseline assessment or 3 and 6-month follow-ups. Five studies [28,31,35,36,37] in adults reported no significant difference in daily carbohydrate intakes (g/day or % energy/day), with one study in adults [37] also reporting that carbohydrate intake was not significantly associated with higher odds of FA or YFAS symptom scores. In children and adolescents, significantly higher intakes of total sugar (g/day, *n* = 1 study [38]), added sugar (g/day or tsp/day, *n* = 2 studies [30,38]) and fructose (g/day, *n* = 1 study [30]) were found in FA compared to NFA; and weak positive associations (*n* = 1 study [38]) reported between YFAS-C symptom scores and total sugar (rs = 0.16, *p* = 0.03), and added teaspoons of sugar (rs = 0.18, *p* = 0.01). Of the three studies in adults [34,35,37], only one study [35] reported significantly higher intakes of sugar (g/kg BW) in FA compared to NFA. *Fats*: Eleven studies assessed total fat intake, with eight of these studies also assessing individual dietary fats. Nine studies [28,33,34,35,36,37,39], including one study in children [30] and one study in adolescents [38], reported significantly higher intakes of total fat (g/day or % energy/day) in FA compared to NFA. In two studies this finding was significant in females only [28,39]. Two studies in adults [31,41] reported no significant difference in daily total fat intake (g/day). In children and adolescents with FA, compared to NFA, significantly higher intakes of trans fat (*n* = 2 studies [30,38]) and saturated fat (*n* = 1 study [38]) were reported. In six studies of adults with FA, compared to NFA, significantly higher intakes of saturated fats (*n* = 3 studies [28,34,35]), monounsaturated fats (*n* = 4 studies [28,34,35,37]), polyunsaturated fats (*n* = 3 studies [28,34,35]) and trans fat (*n* = 1 study [35]) were reported. Odds of FA diagnosis increased with higher intakes of total fat [AOR 1.11 (95% CI: 1.04, 1.18); *p* < 0.001] and monosaturated fat [AOR 1.22 (95% CI: 1.08, 1.38); *p* < 0.001] in one study [37]. Higher YFAS scores were significantly associated with higher total fat intake (*n* = 1, d = 0.16 [37]; *n* = 1, r = 0.20 and r = 0.23, males and females, respectively [39]) and saturated fat intakes in adults in two studies (*n* = 1, d = 0.16 [37]); and higher YFAS-C scores were associated with higher intakes of total fat (rs = 0.26), saturated fat (rs = 0.25) and trans fat (rs = 0.31) in adolescents [38].

#### 3.6.3. Micronutrient Intakes

A range of micronutrients were assessed across six studies. Some significant differences were reported (Table 3), although findings were not consistent across studies. Of particular interest, significantly higher daily sodium intakes (mg/day or mg/kg BW) in FA, compared to NFA, were reported in two studies [30,35], while two further studies [31,37] reported no significant difference. 

#### 3.6.4. Food Items/Food Groups

Addictive eating was most commonly associated with foods high in a combination of fats and sugars. Although food descriptors varied between studies, of the four studies in adults assessing food items or food groups, significantly higher intakes of confectionary (e.g., candy, chocolate; *n* = 3 out of 4 studies [29,37,42]), baked sweet products (e.g., cookies, cakes; *n* = 4 out of 4 studies [29,37,39,42]), savoury snack foods (e.g., potato chips, crackers, popcorn, pretzels; *n* = 3 out of 3 studies [29,37,42]), take out/fast food (e.g., hamburgers, pizza, French fries; *n* = 3 out of 3 studies [29,37,42]), and red/processed meats (*n* = 2 out of 2 studies [39,42]) were reported in FA compared to NFA. 

Odds of FA were strongest among those consuming confectionaries daily or 5–6 times per week (odds ratios ranged from 1.06 to 2.4 [29,37,42]), with one study [29] reporting when confectionaries were consumed two or more times a day the odds of severe FA were 7.1 times higher. Similarly, with increasing frequency of intakes of savoury snack foods and take out/fast food the odds of FA increased significantly (ORs ranged from 1.05 to 2.49). One study [42] reported odds ratios for a variety of baked sweet products. Interestingly, increased odds of FA were observed with store-bought cookies only, while decreased odds were observed for homemade cookies. 

When females and males were compared (*n* = 1 study [39]), females with FA had significantly higher daily intakes of cakes, cookies and biscuits than females without FA, whereas in males with FA intake of cakes, cookies and biscuits was significantly lower than males without FA. In adults, consumption of sugar-sweetened beverages (i.e., soft drinks) was associated with higher odds of FA in one study [OR 1.36 (95% CI 1.07, 1.32); *p* = 0.011] [29]. In contrast, another study [42] reported sugar-sweetened beverages were inversely associated with FA [>once a month: OR 0.70 (95% CI 0.64–0.76), and ≥5–6/week: OR 0.56 (95% CI 0.52–0.61); *p* < 0.001], while low-calorie beverages, with and without caffeine, were positively associated with FA. In children (*n* = 1 study [30]), no significant association was found between soft drink consumption and FA. 

Similar to studies in adults, foods significantly associated with FA in children and adolescents (*n* = 2 studies [30,32]), included cookies/biscuits [AOR = 4.19 (95% CI 1.32, 13.26); *p* = 0.015] [30], French fries [≥1–2 times/week: OR 2.29 (95% CI 0.81, 6.50); *p* = 0.007] [32], hamburgers [≥1–2 times/week: OR 1.53 (95% CI 0.56, 4.21); *p* = 0.106] [32], and sausages [AOR = 11.77 (95% CI 1.29, 107.42); *p* = 0.029] [30]. In a sample of 100 children and adolescents aged 10–18 years [32], the foods reported as ‘most addictive’ were chocolate by 70% of participants, carbonated beverages (59%), ice cream (58%), French fries (57%), white bread (55%), rice (53%), candy (50%), chips (48%) and pasta (43%); with a higher number of girls reporting chocolate to be more ‘addictive’ than boys (*p* < 0.05). In children (*n* = 1 study [30]), while daily energy, macronutrient and micronutrient intakes from ultra-processed foods were reported to be significantly higher in FA, compared to NFA, there was no significant difference in intakes of unprocessed or minimally processed foods.

Across the four studies in adults [29,37,39,42], significantly lower intakes of fruit and/or vegetables (*n* = 2 studies [29,42]), grain foods (*n* = 2 studies [29,37,42]) and breakfast cereals (*n* = 1 study [37]) were reported in FA compared to NFA. Consumption of wholegrain foods (*n* = 1 study [37]), fruit (*n* = 1 study [42]) and vegetables (*n* = 2 studies [29,42]) reduced the likelihood of FA, with one study [29] reporting that each extra unit of vegetable consumption decreased the odds of FA by a factor of 0.8. 

#### 3.6.5. Overall Dietary Quality/Patterns

Two studies [37,40] reported there was no significant difference in diet quality scores between FA and NFA, but individuals with FA had a significantly higher daily energy intake from non-core foods (i.e., energy dense, nutrient poor foods), and a significantly lower daily energy intake from core foods, than NFA. One study reported FA were less likely to consume breakfast every day [29].

## 4. Discussion

To the authors knowledge, this review is the first to comprehensively synthesise the food and nutrient intakes, dietary profiles, and patterns associated with addictive eating. Fifteen studies, primarily conducted in adult populations (*n* = 12), were included in the current review, highlighting that there is limited research within this area, particularly within children and adolescents. The majority of studies assessed energy and/or nutrient intakes and less often food intakes or overall dietary patterns. Although findings across studies were heterogeneous, in general, energy, carbohydrate and fat (total, saturated, polyunsaturated and monounsaturated fat) intakes were significantly higher in adults and children with a YFAS defined FA diagnosis compared to those without. However, these studies were not directly comparable given the differences in dietary assessment tools used and inconsistencies across included studies with respect to reporting outcomes. Significantly higher intakes of sugar (total and added sugar) were more often reported in adolescents and children with FA, than adults with FA. Consistent with previous narrative review [8], the foods most commonly associated with addictive eating were foods with a combination of fat and refined carbohydrates, including confectionary, baked sweet products, savoury snack foods and fast foods, with higher intakes reported in adults and children with FA compared to those without FA. Further, lower intakes of wholegrains, fruits and vegetables were reported in adults and children with FA compared to those without FA. Therefore, the lack of consistency in dietary intakes suggests further investigation into the behavioural aspects of additive eating is warranted [13]. 

While it is important to consider individual nutrient components of the diet to examine the effects on addictive eating outcomes, consideration of complete dietary patterns is essential to capture complex overeating behaviours. In the current review, no significant difference in diet quality scores were found between individuals with and without FA (*n* = 2 studies). However, overall higher intakes of non-core foods (i.e., energy-dense, nutrient poor foods) or highly processed foods and lower intakes of core foods (i.e., nutrient-dense foods, such as fruits and vegetables) were reported in individuals with FA compared to those without. Likewise, a more recent study found poorer diet quality in pregnant females displaying addictive eating tendencies, but not during the postpartum period [43]. Addictive eating in adults and children was most commonly associated with processed foods high in a combination of fats and carbohydrates (e.g., candy, chocolate, cookies, cakes, potato chips, popcorn, pretzels, hamburgers, pizza and French fries). Described less often in the literature is the association between with sugar sweetened beverages and FA. In the current review, no association was found in children and one study reported a positive association in adults [29]. In contrast, another study in a large sample of women, found sugar sweetened beverage consumption was inversely associated with FA and low-calorie beverage consumption was positively associated with FA [42]. The study authors suggest that individuals with FA may substitute sugar sweetened beverages with “diet” beverages. Interestingly, this study also found increased odds of FA were observed with intakes of store-bought cookies, while decreased odds were observed for homemade cookies. The lack of consistent evidence for sugar addiction is consistent with previous reviews [44]. 

The findings of this review are consistent with prior food addiction studies [15,45,46] assessing individual’s subjective experience with foods they consider to be ‘addictive’ or associated with out-of-control eating. For example, Schulte al. 2015 [15] found in two independent samples (*n* = 120 and *n* = 384 participants, respectively), the foods most frequently associated with addictive-like eating behaviours were highly processed foods, with added amounts of fat and/or refined carbohydrates, and a high glycaemic load (e.g., chocolate, ice cream, French fries, pizza, cookie, potato chips, cheeseburger). Similarly, Schulte et al. 2017 [46] observed, in a sample of 501 participants, highly processed foods (e.g., muffin, pizza, cheeseburger) compared to minimally processed foods (e.g., fruits, vegetables, meats) were most associated with all behavioural indicators of addictive-like eating (loss of control, craving, liking and pleasure). It is important to note that the majority of included studies were conducted in Westernised countries. Therefore, self-identified ‘addictive’ foods reported in this review may not be representative of other countries due to differences in food supply, food access, income and sociocultural values associated with food. Future research is warranted across a broad range of countries and food systems to determine similarities and differences in self-perceived addictive foods and associated dietary intakes. 

Although there was some heterogeneity in the dietary intakes associated with FA across included studies, typically, processed foods with a combination of fat and refined carbohydrates were reported to be associated with FA. Therefore, from a clinical perspective, it would be prudent to address intakes of these foods, working towards improving overall diet quality. Addressing diet quality is of particular interest in the context of nutritional psychiatry given the considerable comorbidity between FA and other mental health conditions. In addition, given the differences in dietary intakes and those foods self-identified as addictive, individualised dietary advice is warranted. Further, given the current review found no clear differences in dietary intakes or patterns between individuals with and without FA, it may be worth considering the behavioural aspects of addictive-like eating in treatment plans as opposed to targeting specific nutrients.

While prior studies have reported that specific types of processed foods, such as those found in the current review, are associated with higher risk of addictive eating, few have used a standardised approach to classify processed foods. In the current review, only one study used a standardised system (i.e., NOVA) to classify foods according to the degree of processing (unprocessed/minimally processed foods vs. ultra-processed foods). Daily energy, macronutrient and micronutrient intakes from ultra-processed foods were reported to be significantly higher in children with FA compared to those without. Currently, no research has directly compared whether the degree of processing or nutrient content is more strongly related to increased risk of FA. Such research is needed to determine whether a focus on processing is more advantageous than other food classifications or measures, such as dietary quality indexes or nutrient profiling scores, for uncovering relationships between diet and addictive eating. At present, there are no standardised measurement systems to quantitatively define foods thought to be ‘addictive’ or ‘rewarding’ by their nutrient contents, and researchers rely on descriptive definitions. For example, ‘hyperpalatable’, ‘reinforcing’, ‘discretionary choice’, ‘fast foods’, ‘energy-dense, nutrient poor’ or ‘junk foods’. Recently, Fazzino et al. [47] sought to define quantitative criteria for ‘hyperpalatable foods’. A quantitative definition of ‘addictive’ foods would allow a more standardised approach to dietary assessment in FA. This will also be useful to identify whether associations between FA and the ingredients (e.g., flour) contained within homemade or commercial versions of foods (e.g., cookies) depend on ingredients used or the level of processing. Given that global food systems have undergone marked changes in availability, affordability, and marketing of highly processed foods due to advances in food processing and technology, it would seem further research in relation to FA is warranted.

All the included studies used the YFAS to assess addictive eating and provide a ‘FA diagnosis’. The prevalence of FA found across studies was similar to that found in other systematic reviews [5,6,7], with higher prevalence in individuals with higher weight status and in those with mental health comorbidities. It must be noted that not all individuals with addictive eating behaviours will meet the criteria for “food addiction” due to the self-perceived nature of the clinical impairment or distress criterion required for a YFAS FA diagnosis. It has been suggested that overeating may best be viewed along a dimension reflecting degrees of severity and compulsiveness, and that the high end of the continuum marks the endorsement of clinically significant impairment and distress [48]. The current review found higher scores in FA symptomatology were positively associated with intakes of carbohydrate and fat intakes in adults and children, and sugar intake in children. This highlights that it may be worthwhile considering symptom scores, irrespective of FA diagnosis, to interpret findings from the YFAS when dealing with overeating behaviours. While a recent review highlights that the many self-report measures developed to understand the phenomena underpinning overeating correlate strongly [49], it should be acknowledged that the YFAS remains a common self-reported tool. However, there are documented differences in self-perceived food addiction (47%) [50] and prevalence as identified using a self-report tool such as the YFAS (15–20%) [5], as well as those who believe certain foods to possess addictive qualities (86%) [51]. Therefore, the findings of this review should be interpreted in the context of this compared to other fields of research such as preclinical and human drug and alcohol addiction research which have more well-established, clear and objective diagnostic criteria. In addition, future research may consider the use of other FA tools (e.g., the Addiction-like Eating Behaviour Scale) to determine if the dietary intakes associated with the YFAS, as identified in this review, are consistent across tools. 

All the included studies used conventional dietary assessment methods to assess dietary outcomes. Food Frequency Questionnaires were the most common tool used (*n* = 10 studies) and the remaining studies used methods (i.e., 24 h recalls, food diaries/records or standardised survey questions) that assessed dietary outcomes over much shorter reporting periods. Findings from this review suggest that validation of dietary-assessment methods or tools within populations with FA is needed, and closer consideration given to reporting the methods used to assess dietary intake (e.g., classification system of foods/food groups, details of the tool used, tool administration method and reporting periods). Further, adjustments for measurement error (e.g., adjustments for misreporting such as the use of the Goldberg cutoffs [52]) could be made in future studies. Assessment of usual/habitual intake over longer time frames is of particular interest to determine day-to-day, and weekday-to-weekend day, fluctuations in intake versus the chronic over consumption of specific foods. This may be important to further our understanding in the context of the overlap that FA presents with Binge Eating Disorder (see [29,53]).

This review has several strengths. A broad search using a range of terms was conducted across six databases to provide a description of dietary intakes associated with addictive eating. A range of nutrient and dietary outcomes were considered, in samples across a wide age range (i.e., children, adolescents and adults). There are also limitations to the current review. Firstly, only studies published in English language were included. Secondly, the majority of participants in the identified studies were female, and most samples consisted of higher proportion of participants with overweight/obesity compared to participants with healthy weight, which may limit the generalisability of the findings. As FA is not synonymous with higher weight status, it will be important to assess a range of weight statuses in future research. Five studies included participants with health conditions/diagnoses involving direct medical treatment (i.e., individuals with schizophrenia or patients undergoing bariatric surgery), or participants seeking weight loss treatment, and it is unclear if any alterations in dietary patterns had commenced prior to individuals enrolling in these studies. Only three studies of children/adolescents were retrieved by the search strategy. Further research in younger age groups is warranted. Lastly, studies may not be comparable due to the highly variable measurement metrics and food/food group descriptors used across the included studies, which also limited the opportunity to meta-analyse dietary outcomes. Additionally, misreporting of dietary intake (over- and underreporting) was not assessed in the majority of studies. Given this is one of the main sources of error in dietary assessment the validity of the data collected, and conclusions drawn may be affected. Therefore, the results need to be interpreted in the context of these limitations. 

## 5. Conclusions

Differences in dietary intakes were observed according to addictive eating including macronutrients, specific foods, and dietary intakes. However, there was considerable heterogeneity due to differences in samples recruited, diet outcomes assessed, and dietary assessment methods used. Due to the small number of studies retrieved by the review, it is difficult to specifically conclude if any specific foods, nutrients or dietary patterns facilitate an addictive process. However, this review highlights the need for further high-quality study designs including rigorous dietary assessment methodology to elucidate any potential associations. Future treatment approaches should incorporate individualised dietary advice targeting foods high in fat and refined carbohydrates.

## Figures and Tables

**Table 1:** PICOS criteria for studies assessing dietary intakes and patterns associated with addictive eating in adolescents and adults.

PICOS Criteria	Description
Population	Males and females of all ages with and without addictive eating
Intervention	Studies that evaluated dietary intake by FFQ, 24 h recall methods, food records or similar instruments
Comparators	Studies using a control group of individuals without addictive eating or no comparator
Outcomes	Addictive eating status; food and nutrient intakes, dietary profiles, and dietary patterns
Setting	All settings (observational or experimental study designs)

**Table 2:** Description of included studies.

Author, Year, Country	Type of Study	Number of Participants	Population Studied	Participant Characteristics–Mean age ± SD; % Female; Mean BMI/BMIz ± SD (Range); Ethnicity	Addictive Eating Assessment Measures— Tool Used; Administration Method	Symptom Score (SS) or Diagnosis (D) Used	Dietary Outcomes Assessed	Dietary Assessment—Measure Used; Administration Method; Reporting Period; Analysis FCD(s) and/or Software; Adjustments for Misreporting
Ayaz [28], 2018, Turkey	Cross- sectional	851	Healthy adults living in Ankara aged 19–65 y	Age: 34.6 ± 12.8 y; 57.7% F; Median (min-max) BMI in NFA 23.7 kg/m^2^ (15.4–43.8) and in FA 26.8 kg/m^2^ (17.0–47.6), 9.4% underweight, 47.2% healthy weight, 27.7% overweight, 15.6% obesity; ethnicity NR	YFAS, 25-item, Turkish version; interview conducted by intern dietitian	D	Energy, macronutrients and micronutrient intake (protein, total fat, SFA, MUFA, PUFA, cholesterol, CHO, fibre, K, Ca, Mg, Fe, Zn, folic acid; Vit A, B1, B2, B12, C and E)	24 h dietary recall; via interview with dietitian; NR; photographic atlas and BeBIS-6.1 (Nutrition Information Systems Software); misreporters excluded from analysis using Goldberg cut-offs
Ben Porat [41], 2020, Israel	Prospective cohort study (3, 6 and 12-month post-operative follow up)	54(*n* = 54, 3, 6 and 12-month follow up)	Females with BMI ≥ 40 kg/m^2^ or BMI ≥ 35 kg/m^2^ with comorbidities presenting for sleeve gastrectomy surgery, aged 18–65 y	Age: NFA 32.9 ± 11.4 y, FA 30.8 ± 10.8 y; 100% F; BMI in NFA 44.9 ± 4.4 kg/m^2^ and in FA 44.9 ± 5.6 kg/m^2^; 100% obesity; ethnicity NR	YFAS, 25-item, Hebrew and Arab translations; NR	D + SS	Energy, protein and CHO intake (fat intake at 12-month follow-up only)	Food diary; NR; 3 days (unclear if weekend and/or week days assessed, or average of 3 days used); Zameret Israeli nutritional software; misreporting not assessed
Burrows [29], 2017, Australia	Cross-sectional	1344	Adults living in Australia aged ≥ 18–91 y	Age of 39.8 ± 13.1 y; 75.7% F; BMI 27.7 ± 9.5 kg/m^2^, 45.2% healthy weight, 26.0% overweight, 28.8% obesity; 2.0% indigenous	YFAS 2.0, 35-item; online survey	D + SS	Core^a^ and non-core^b^ food intake	Standardised questions derived from the New South Wales Health Survey, 20-item; online survey; 7 days; N/A; misreporting not assessed
Filgueras [30], 2019, Brazil	Cross-sectional	139	Low-income school children with overweight or obesity enrolled in a longitudinal study, aged 9–11 y	Age 9.6 ± 0.7 y; 54% F; BMI z-score 1.9 ± 0.7, 56.1% overweight, 36.7% obesity, 6.6% severe obesity; ethnicity NR	YFAS-C, 25-item; paper-based survey, interviewer assisted	D + SS	Energy, macronutrient and micronutrient intake (total protein, animal protein, vegetable protein, total fat, trans fat CHO, total sugar, added sugar, fructose, fibre, Na) from total diet, unprocessed/minimally processed foods and ultra-processed foods	Brazilian FFQ, semi-quantitative, 88 items (41 ultra-processed items, 12 processed items, 35 unprocessed/minimally processed items), portion sizes estimated using a photographic manual; interviewer administered; NR; Nutrition Data System Research (NDS-R version 2014) and TACO (Brazilian FCD), NOVA for processed food classification; misreporting not assessed
Grammatikopolou [31], 2018, Greece	Cross- sectional	176	Undergraduate students from Department of Nutrition and Dietetics in Thessaloniki, Greece, aged 18–40 y	Age 21.7 ± 1.9 y; 79.5% F; BMI in non-orthorexic (*n* = 56) 21.5 ± 3.5 kg/m^2^ and in orthorexic (*n* = 120) 22.3 ± 2.9 kg/m^2^; ethnicity NR	mYFAS, 9-item; paper-based survey	D + SS	Energy, macronutrient (protein, total fat, SFA, MUFA, PUFA, trans fat, CHO, fibre) and Na intake	Food diary; online; 3 days (unclear if weekend and/or week days assessed, or average of 3 days used); ESHA’s Food Processor ^®^ Nutrition Analysis software; nutrient intake adjusted for energy consumption using the residual method
Keser [32], 2015, Turkey	Cross- sectional	100	Children and adolescents with overweight and obesity attending a paediatric outpatient clinic, aged 10–18 y	Age: NFA 13.9 ±1.96 y, FA 14.6 ± 2.07 y; 63% F; BMI-z score in NFA 2.6 ± 1.18 (0.6–6.6) and in FA 2.6 ± 0.65 (1.0–4.1), 20% overweight (>+1 SD), 80% obesity (>+2 SD); ethnicity NR	YFAS, 25-item; NR, completed by participant not parent	D	Energy dense food/beverage intake; most addictive foods reported	FFQ (Not specified. Other details of diet intake method may be available in cited reference, however English publication unavailable); NR; NR; N/A; misreporting not assessed
Kucukerdonmez [33], 2017, Turkey	Cross-sectional	104	Adults with DSM-5 diagnosed schizophrenia, aged 20–40 y	Age: 39.4 ±10.78 y; 60.8% F; BMI 28.5 ± 6.13 kg/m^2^, 69.3% overweight and obesity; ethnicity NR	YFAS, 25-item; NR	D + SS	Energy and macronutrient intake (protein, fat, SFA, MUFA, PUFA, CHO, fibre)	Food record; NR; 3 days (unclear if weekend and/or week days assessed, or average of 3 days used); Nutrition Information Systems Package; misreporting not assessed
Lemeshow [42], 2017, USA	Prospective cohort study (Cross-sectional analyses)	123,688 (NHS, *n* = 58,625; NHSII, *n* = 65,063)	Female nurses participating in the Nurses’ Health Study (NHS) and Nurses’ Health Study II (NHSII)	Age range 45–87 y (mean age NR); 100% F; Mean BMI NR, BMI categories (*n* = 122,316) 43.4% healthy weight, 31.6% overweight, 25.0% obesity; 92.6% Caucasian, 1.0% African American, 1.0% Asian, 0.9% Hispanic, 4.4% Other/Unknown	mYFAS, 9-items; NR. FA data collected in 2008 (NHS) and 2009 (NHSII).	D	Energy intake, intake of food groups (Red/processed meats, snacks, sweets and desserts, refined grains, fruits and vegetables, no/low fat snacks and sweets, no/low fat dairy, low calorie beverages, sugar sweetened beverages) and 39 positively reinforcing food items in contrast to non-reinforcing food items (identified from published research)	Semi-quantitative FFQ, 131-item; NR diet data collected in 2006 (NHS) and 2007 (NHSII); NR; Method for analysis of energy intake NR; misreporting not assessed
Moghaddam [34], 2019, Iran	Cross- sectional	244	Females with obesity attending a weight management clinic, aged 18–60 y	Age 39 ± 10 y; 100% F; Median BMI (IQR) 36.37 (30.0–78.3); ethnicity NR	YFAS, 25-item, Iranian version; NR	D	Energy and macronutrient intake (protein, fat, SFA MUFA PUFA, trans fat, CHO, sucrose)	FFQ, semi-quantitative, 147 items; interviewer administered; 12 months; USDA FCD, Nutritionist IV software; misreporting not assessed
Pedram [36], 2013, Canada	Cross- sectional	652	Third generation healthy adults living in Canadian province of Newfoundland and Labrador, aged > 19–90 y	Age: 44.3 ± 12.9 y; 63.7% F; BMI 27.4 ± 5.4 kg/m^2^ (17.05–54.2), 38.2% underweight/normal weight, 61.8%. overweight or obese; ethnicity NR	YFAS, 25-item; NR	D	Macronutrient intake (protein, fat, CHO, fibre)	Willett FFQ, semi-quantitative, 61 items; NR; 12 months; NutriBase Clinical Nutrition Manager software (version 9); misreporting not assessed
Pedram, 2015 [35], Canada	Cross- sectional (subsample analysis of Pedram, 2013)	58	Adults with overweight and obesity with YFAS defined food addiction, and non–food addicted matched controls, aged > 19 y	Age NFA 42 ± 8.9 y, FA 42.5 ± 9.4 y; 82.8% F; BMI in NFA 32 ± 4.42 kg/m^2^ and in FA 32.5 ± 6 kg/m^2^; ethnicity NR	YFAS, 25-item; NR	D	Energy, macronutrient and micronutrient intake (protein, total fat, SFA, MUFA, PUFA, trans fat, CHO, sugar, omega 3 and 6, Na, K, Ca, Se; vitamins B1, D, E and K)	Willett FFQ, semi-quantitative, 61 items; NR; 12 months; NutriBase Clinical Nutrition Manager software (version 9); misreporting not assessed
Pursey [37], 2015, Australia	Cross- sectional	462	Adults living in Australia aged 18–35 y	Age 25.1 ± 4.0 y; 86.0% F; BMI: 23.2 ± 4.5 kg/m^2^ (15.9–54.0), 5.4% underweight, 72.9% healthy weight, 13.9% overweight, 7.8% obesity; ethnicity NR	YFAS, 25-item; online survey	D + SS	Energy, macronutrient and micronutrient intake (protein, total fat, SFA, MUFA, PUFA, cholesterol, CHO, sugars, fibre, alcohol, folate, Na, K, Mg. Ca, P, Fe, Zn; vitamins A, B1, B2, B3, and C); energy from food groups (vegetables, fruit, meat, grains, dairy, sweet drink, savoury packaged snacks, candy, baked sweet products, take-out, breakfast cereal); energy from core ^a^ and non-core foods ^b^; diet quality score	Australian Eating Survey FFQ semi-quantitative, 120 items, and Australian Recommended Food Score (ARFS) derived from subset of 70 items (scores range from 0–73, higher scores reflect overall higher nutritional quality of usual eating pattern); online survey; 6 months; AUSNUT Australian FCD
Schulte [38], 2018, USA	Intervention study (baseline data reported)	181	Adolescents with obesity participating in weight management program, 12–16 y	Age 13.75 ± 1.35 y; 67% F; BMI: 38.2 ± 7.5 kg/m^2^ (25.7–60.5); 100% African American	YFAS-C, 25-item; paper-based form, completed by participant not parent	D + SS	Energy, total fat, SFA, trans fat, CHO, total sugar and added sugar intake	The Block Kids FFQ; paper-based form; one week; NutritionQuest assessment and analysis service
Sengor [39], 2020, Turkey	Cross- sectional	370	University students aged 18–25 y	Age 18–25 years (mean age NR); 56.8% F; BMI NR; ethnicity NR	YFAS, 25-item. Turkish version; via interview	D + SS	Energy, macronutrient and micronutrient intake (protein, fat, cholesterol, CHO and fibre, K, Ca, Mg, P, Fe, Zn, folic acid; and vitamins A, B1, B2, B3, B6, B12, C and E); and intake of food items/groups (milk and dairy products; bread and grains; oily seeds; meat and egg; sausage; vegetables; potato, starch and mushroom; fruits; sweets; cakes, cookies and biscuits; oil and fat)	FFQ (not specified), 54 food items, Turkish adaptation; paper-based form; one month; BeBIS-7.2 (Nutrition Information Systems Software)
Skinner [40], 2019, Australia	RCT (baseline data reported)	18	Healthy females aged 18–85 y	Age 43.0 ± 16.5 y; 100.0% F; BMI in NFA 25.64 ± 2.84 kg/m^2^ (21.22–29.97) and in FA 33.55 ± 6.02 kg/m^2^ (25.66–41.48), 33.3% healthy weight, 50.0% overweight, 16.7% obesity; ethnicity NR	YFAS 2.0, 35-item; online survey	D + SS	Energy from core ^a^ and non-core ^b^ foods, diet quality score	Australian Eating Survey FFQ semi-quantitative, 120 items, and Australian Recommended Food Score (ARFS) derived from subset of 70 items (scores range from 0–73, higher scores reflect overall higher nutritional quality of usual eating pattern); online survey; 6 months; AUSNUT Australian FCD

BMI, Body Mass Index; CHO, carbohydrate; Ca, calcium; FA, food addicted; Fe, iron; FFQ, Food Frequency Questionnaire; K, potassium; Mg, magnesium; MUFA, monounsaturated fatty acids; mYFAS, modified Yale Food Addiction Scale; Na, sodium; N/A, not applicable; NFA, non-food addicted; NR, not reported; P, potassium; PUFA, polyunsaturated fatty acids; RCT, randomised controlled trial; SD, standard deviation; Se, selenium; SFA, saturated fatty acids; YFAS, Yale Food Addiction Scale; YFAS 2.0, Yale Food Addiction Scale (version 2.0); YFAS-C, Yale Food Addiction Scale for Children, Zn, zinc. ^a^ Core foods include fruit vegetables, dairy, meat/protein and carbohydrate/grain-based foods. ^b^ Non-core foods include foods with added or high amounts of salt, sugar and/or fat.

**Table 3:** Outcomes of included studies.

Author, Year, Country	Prevalence of FA by Diagnosis	YFAS Symptoms Mean ± SD (Range or 95% CI)	Relationship/Association between YFAS and Diet	Study Limitations
Ayaz [28], 2018, Turkey	11.4%	NR	Significantly higher daily intakes of energy, protein and fat, SFA, PUFA, MUFA, cholesterol Vitamin A, Vitamin E, Vitamin B12, Mg, Fe, Zn in females with FA compared to NFA. No significant difference in energy, macronutrient or micronutrient intakes in males with FA compared to NFA.	Predominantly younger adults 19–39 y
Ben Porat [41], 2020, Israel	40.7%	Median and IQR NFA = 2.0 (1.0, 3.0) FA = 5.5 (4.0, 7.0)	No significant differences in daily intakes of energy, protein or CHO intake between FA and NFA at each follow-up timepoint, according to baseline FA diagnosis. Prevalence of FA decreased from baseline to 29.3% at 12-month post-surgery. In those with a FA diagnosis at 12-month post-surgery, significantly higher daily intake of CHO, compared to NFA. No significant difference in protein and fat intakes.	Specific population of adults undergoing bariatric surgery. Fat intake not reported according to baseline FA diagnosis.
Burrows [29], 2017, Australia	22.2% (FA severity: 3.1% Mild, 11.8% Moderate, 85.1% Severe)	8.1 ± 2.6 (baseline)	FA reported higher intakes of confectionary, fast food, snack foods, hot chips, potato crisps, soft drinks and lower intakes of core foods ^a^, such as fruits and vegetables, compared to NFA. FA were less likely to consume breakfast every day. FA who ate confectionaries daily or 5–6 times per week had 2.4 times the odds of severe FA, while those who ate confectionaries two or more times a day had 7.1 times the odds of severe FA. Vegetable intake reduced the likelihood of severe FA, with each extra unit of vegetable consumption decreasing the odds by a factor of 0.8.	Predominantly younger adults 18–54 y. Predominantly female sample.
Filgueras [30], 2019, Brazil	24.0%	3.0 (95% CI: 3.1 to 3.8)	Significantly lower daily intake of fibre, and higher daily intakes of protein (total, vegetable and animal), fat (total and trans fat), CHO, sugar (total, added and fructose) and sodium in FA compared NFA. Significantly higher daily energy, macronutrient and micronutrient intakes from ultra-processed foods in FA compared to NFA. No significant difference in daily energy, macronutrient and micronutrient intakes from unprocessed/minimally processed foods in FA compared to NFA. Ultra-processed foods positively associated with FA were cookies/biscuits and sausages.	Children are participating in a school-based intervention, and it is not clear if FA or dietary intake was measured at baseline or during the intervention.
Grammatikopolou [31], 2018, Greece	4.5%	1.4 ± 1.0	No significant difference in energy, macronutrient or micronutrient intakes between FA and NFA.	Specific population of nutrition and dietetic students, may have been motivated by health; predominantly female, small number of FA
Keser [32], 2015, Turkey	71.0%	NR	Foods high in CHO and fats perceived to be the most addictive. Consumption of French fries ≥ 1–2 times per week associated with a 2.3-fold increase in FA risk.	Unclear if validated FFQ used. Intake patterns for NFA not reported and difference in mean intakes between FA and NFA not assessed.
Kucukerdonmez [33], 2017, Turkey	60.6%	3.5 ± 1.7	Significantly higher daily intakes of energy, CHO, fibre, total fat and PUFA in FA compared to NFA.	Specific population of adults with schizophrenia
Lemeshow [42], 2017, USA	2.5% and 8.0%	NR	FA was associated with higher intakes of foods hypothesised to be positively reinforcing (i.e., foods containing high amounts of refined CHO and fat) such as fast foods, snacks, desserts, fast food and candy. Odds of FA were strongest among those consuming 5+ servings/week (compared with <1 serving/month) of hamburgers, French fries and pizza. Consumption of red/processed meat, low/no fat snacks/desserts, and low-calorie beverages was positively associated with FA. Consumption of refined grains, sugar-sweetened beverages and fruit and vegetables was inversely associated with FA.	Female nurses, predominantly Caucasian, and all >45 y
Moghaddam [34], 2019, Iran	27.9%	NR	Significantly higher intakes of energy, protein, CHO, total fat, SFA, MUFA, PUFA and cholesterol in FA compared to NFA. No significant difference in intakes of sucrose and trans fat between FA and NFA.	Specific population of females with obesity attending a weight management clinic
Pedram [36], 2013, Canada	5.4%	NR	Significant higher percent energy from protein and fat in FA compared to NFA. No significant difference in intakes of protein, CHO and fat when expressed as gram per kg of body weight.	Predominantly female, few nutrients assessed
Pedram [35], 2015, Canada	50.0%	NR	Significantly higher percent energy from fat in FA compared to NFA. When expressed as gram per kg of body weight, FA had significantly higher intakes of energy, CHO, dietary sugar, fat, SFA, trans fat, MUFA, PUFA, omega 3, omega 6, Na, P, Ca, Se, vitamin B1, vitamin D, gamma-tocopherol and dihydrophylloquinone compared to NFA.	Small sample, predominantly female
Pursey [37], 2015, Australia	14.7%	2.4 ± 1.8	Higher YFAS scores associated with higher intakes of energy-dense, nutrient-poor foods (candy, take out and baked sweet products) and lower intakes of nutrient-dense core foods ^a^ (whole-grain products and breakfast cereals). Higher intakes of total fat and MUFA, and lower intakes of wholegrain foods in FA compared to NFA. Odds of FA diagnosis increased with higher intakes of fat and MUFA. Odds of FA diagnosis decreased with higher intakes of wholegrain foods.	Predominantly female, and well-educated, sample
Schulte [38], 2018, USA	9.9%	2.11 ± 1.75	Significantly higher intakes of energy, total fat, trans fat, total CHO, total sugar, and added sugar in FA compared to NFA. Significant positive associations between YFAS-C scores and total calories, fat, SFA, trans fat, total CHO, total sugar, and added sugar.	All African American sample; short reporting duration of dietary intake.
Sengor [39], 2020, Turkey	21.1%	NR	Higher YFAS scores associated with higher intakes of energy, CHO and fat in males and females. Females with FA had higher daily intakes of energy, CHO and fat; and lower intakes of Vit C. No difference in energy, macro- and micronutrient intakes between males with and without FA. Females with FA consumed greater amounts of meat and egg; sausage; and cakes, cookies and biscuits per day compared to females without FA; and males with FA consumed greater amounts of oily seeds; oil and fat; lower amounts of cakes, cookies and biscuits per day compared to males without FA.	Mean age and weight status not reported. Unclear if validated FFQ used.
Skinner [40], 2019, Australia	33.3%	1.1 ± 1.2	Significantly lower intakes of core ^a^ foods and higher intakes of non-core ^b^ foods in FA compared to NFA. No significant difference in diet quality score between FA and NFA.	Small sample, females only, large age range. Significant difference in BMI between FA and NFA groups. Subgroups of core and non-core foods not reported.

ARFS, Australian Recommended Food Score, BMI, Body Mass Index; Ca, calcium; CHO, carbohydrate; CI, confidence interval, E, energy; EI, energy intake; FA, food addicted; Fe, iron; FFQ, Food Frequency Questionnaire; IQR, Interquartile Range; K, potassium; Mg, magnesium; MUFA, monounsaturated fatty acids; mYFAS, modified Yale Food Addiction Scale; Na, sodium; N/A, not applicable; NFA, non-food addicted; NR, not reported; P, potassium; PUFA, polyunsaturated fatty acids; RCT, randomised controlled trial; SD, standard deviation; Se, selenium; SFA, saturated fatty acids; YFAS, Yale Food Addiction Scale; YFAS-C, Yale Food Addiction Scale for Children, Zn, zinc. ^a^ Core foods include fruit vegetables, dairy, meat/protein and carbohydrate/grain-based foods. ^b^ Non-core foods include discretionary choice foods with added or high amounts of salt, sugar and/or fat.

## Supplementary Materials

The following are available online at https://www.mdpi.com/article/10.3390/nu14010164/s1, Table S1. Example of Search Strategy (November 2020), Table S2. Quality of Included Studies, Table S3. Dietary Outcomes of Included Studies.
