# Supplementary material for: The Relationship between Addictive Eating and Dietary Intake: A Systematic Review
Source: Nutrients. 2021 Dec 30;14(1):164. doi: 10.3390/nu14010164 (PMC8747081; doi:10.3390/nu14010164)
Supplement: Supplementary file 1 [file nutrients-14-00164-s001.zip › nutrients-1505518-supplementary.pdf]

Supporting Information:

Article type: Review

Title: The relationship between addictive eating and dietary intake: A systematic review

Authors: Kirrilly M Pursey[1,2], Janelle A Skinner[1,2], Mark Leary[1,2], Tracy L Burrows[1,2]

Author Affiliations:

[1]Nutrition and Dietetics, School of Health Sciences, College of Health, Medicine and Wellbeing, University Drive, University of Newcastle, Callaghan NSW 2308, Australia

[2]Priority Research Centre for Physical Activity and Nutrition, University of Newcastle, University Drive, Callaghan NSW 2308, Australia

***Corresponding Author:** Dr Kirrilly Pursey. Mailing address: School of Health Sciences, University Drive, University of Newcastle, Callaghan, NSW, Australia, 2308. Ph: +610249215514. Email: kirrilly.pursey@newcastle.edu.au

PRISMA-P 2015 Checklist

Section/topic	#	Checklist item	Information reported		Page number(s); comments
			Yes	No	
**ADMINISTRATIVE INFORMATION**					
**Title**					
Identification	1a	Identify the report as a protocol of a systematic review	✓		1
Update	1b	If the protocol is for an update of a previous systematic review, identify as such		✓	N/A
**Registration**	2	If registered, provide the name of the registry (e.g., PROSPERO) and registration number	✓		6
**Authors**					
Contact	3a	Provide name, institutional affiliation, and e-mail address of all protocol authors; provide physical mailing address of corresponding author	✓		1
Contributions	3b	Describe contributions of protocol authors and identify the guarantor of the review	✓		7, 24-25
**Amendments**	4	If the protocol represents an amendment of a previously completed or published protocol, identify as such and list changes; otherwise, state plan for documenting important protocol amendments		✓	N/A
**Support**					
Sources	5a	Indicate sources of financial or other support for the review	✓		24
Sponsor	5b	Provide name for the review funder and/or sponsor			N/A
Role of sponsor/funder	5c	Describe roles of funder(s), sponsor(s), and/or institution(s), if any, in developing the protocol			N/A
**INTRODUCTION**					
**Rationale**	6	Describe the rationale for the review in the context of what is already known	✓		3-5
**Objectives**	7	Provide an explicit statement of the question(s) the review will address with reference to participants, interventions, comparators, and outcomes (PICO)	✓		5
**METHODS**					

Section/topic	#	Checklist item	Information reported		Page number(s); comments
			Yes	No	
**Eligibility criteria**	8	Specify the study characteristics (e.g., PICO, study design, setting, time frame) and report characteristics (e.g., years considered, language, publication status) to be used as criteria for eligibility for the review	✓		6
**Information sources**	9	Describe all intended information sources (e.g., electronic databases, contact with study authors, trial registers, or other grey literature sources) with planned dates of coverage	✓		5, 6
**Search strategy**	10	Present draft of search strategy to be used for at least one electronic database, including planned limits, such that it could be repeated	✓		Suppl.
STUDY RECORDS					
Data management	11a	Describe the mechanism(s) that will be used to manage records and data throughout the review	✓		6, 7
Selection process	11b	State the process that will be used for selecting studies (e.g., two independent reviewers) through each phase of the review (i.e., screening, eligibility, and inclusion in meta-analysis)	✓		6, 7
Data collection process	11c	Describe planned method of extracting data from reports (e.g., piloting forms, done independently, in duplicate), any processes for obtaining and confirming data from investigators	✓		7
**Data items**	12	List and define all variables for which data will be sought (e.g., PICO items, funding sources), any pre-planned data assumptions and simplifications	✓		7
**Outcomes and prioritization**	13	List and define all outcomes for which data will be sought, including prioritization of main and additional outcomes, with rationale	✓		6, 7
**Risk of bias in individual studies**	14	Describe anticipated methods for assessing risk of bias of individual studies, including whether this will be done at the outcome or study level, or both; state how this information will be used in data synthesis	✓		7, 8
DATA					
**Synthesis**	15a	Describe criteria under which study data will be quantitatively synthesized		✓	N/A see pg 7
	15b	If data are appropriate for quantitative synthesis, describe planned summary measures, methods of handling data, and methods of combining data from studies, including any planned exploration of consistency (e.g., I^2, Kendall's tau)			N/A

Section/topic	#	Checklist item	Information reported		Page number(s); comments
			Yes	No	
	15c	Describe any proposed additional analyses (e.g., sensitivity or subgroup analyses, meta-regression)			N/A
	15d	If quantitative synthesis is not appropriate, describe the type of summary planned	✓		7
**Meta-bias(es)**	16	Specify any planned assessment of meta-bias(es) (e.g., publication bias across studies, selective reporting within studies)	✓		N/A
**Confidence in cumulative evidence**	17	Describe how the strength of the body of evidence will be assessed (e.g., GRADE)	✓		N/A

Checklist adapted from Table 3 in: Moher D, Shamseer L, Clarke M, et al. Preferred reporting items for systematic review and meta-analysis protocols (PRISMA-P) 2015 statement. Systematic Reviews. 2015;**4**:1-9.
Available from: http://www.systematicreviewsjournal.com/content/4/1/1 ; http://www.prisma-statement.org/Extensions/Protocols

Table S1. Example of Search Strategy (November 2020)

# ▲	Searches	Results
1	Behavior, Addictive/	10078
2	addiction.mp.	48209
3	Substance-related disorders.mp.	96688
4	Food addiction.mp.	657
5	Yale food addiction scale.mp.	219
6	YFAS.mp.	144
7	(food* adj5 addict*).mp.	882
8	Craving/	1498
9	(eat* adj5 addict*).mp.	561
10	(compulsive adj5 eat*).mp. [mp=title, abstract, original title, name of substance word, subject heading word, floating sub-heading word, keyword heading word, organism supplementary concept word, protocol supplementary concept word, rare disease supplementary concept word, unique identifier, synonyms]	714
11	Food habits.mp.	2113
12	Eating behaviour.mp.	2019
13	Eating behavior.mp.	5162
14	Feeding behavior.mp.	86969
15	Feeding behaviour.mp.	2370
16	Food preferences.mp.	15425
17	Diet/	161077
18	Energy Intake/	41024
19	Nutrients/	2320
20	diet quality.mp.	4201
21	processed food.mp.	1309
22	ultraprocessed food.mp.	20
23	Food/	33449
24	dietary intake.mp.	25253
25	1 or 2 or 3 or 4 or 5 or 6 or 7 or 8 or 9 or 10	137493
26	11 or 12 or 13 or 14 or 15 or 16 or 17 or 18 or 19 or 20 or 21 or 22 or 23 or 24	315718
27	25 and 26	1874
28	limit 27 to (english language and humans)	1302

Table S2. Quality of Included Studies

Study quality and risk of bias assessed using ©2016 Evidence Analysis Manual Academy of Nutrition and Dietetics. Available from: https://www.andeal.org/vault/2440/web/files/2016_April_EA_Manual.pdf

	Relevance Questions				Validity Questions										
	R1	R2	R3	R4	V1	V2	V3	V4	V5	V6	V7	V8	V9	V10	
Author, year	Improved outcomes for the patients/ clients/ population group	Outcome/s that patients/ clients/population group would care about	Study topic relevant to dietetic practice	Feasible intervention procedure	Research question clearly stated	Participant selection free from bias	Study groups comparable	Withdrawals described	Blinding of outcomes and risk factors	Intervention described	Outcomes defined, measures valid	Appropriate statistical analysis	Conclusions supported	Funding bias unlikely	OVERALL rating*
Ayaz, 2018[1]	N/A	Y	Y	N/A	Y	Y	Y	Y	UC	Y	Y	Y	Y	Y	+
Ben Porat, 2020[2]	N/A	Y	Y	N/A	Y	Y	Y	Y	UC	Y	Y	Y	Y	Y	+
Burrows, 2017[3]	N/A	Y	Y	N/A	Y	Y	Y	Y	UC	Y	Y	Y	Y	Y	+
Filgueras, 2019[4]	N/A	Y	Y	N/A	Y	Y	Y	N	Y	Y	Y	Y	Y	Y	+
Grammatikopolou, 2018[5]	N/A	Y	Y	N/A	Y	Y	Y	Y	Y	Y	Y	Y	Y	Y	+
Keser, 2015[6]	N/A	Y	Y	N/A	Y	Y	Y	UC	Y	Y	UC	Y	UC	UC	Ø
Kucukerdonmez, 2017[7]	N/A	Y	Y	N/A	Y	Y	Y	UC	UC	Y	Y	Y	UC	Y	+
Lemeshow, 2017[8]	N/A	Y	Y	N/A	Y	Y	Y	Y	UC	Y	Y	Y	Y	Y	+
Moghaddam, 2019[9]	N/A	Y	Y	N/A	Y	Y	Y	Y	Y	Y	Y	Y	Y	Y	+
Pedram, 2013[10]	N/A	Y	Y	N/A	Y	Y	Y	Y	UC	Y	Y	Y	Y	Y	+

Pedram, 2015[11]	N/A	Y	Y	N/A	Y	Y	Y	Y	Y	Y	Y	Y	Y	Y	+
Pursey, 2015[12]	N/A	Y	Y	N/A	Y	Y	Y	Y	UC	Y	Y	Y	Y	Y	+
Schulte, 2018[13]	N/A	Y	Y	N/A	Y	Y	Y	Y	UC	Y	Y	Y	Y	Y	+
Segnor, 2020[14]	N/A	Y	Y	N/A	Y	Y	Y	UC	UC	Y	UC	Y	UC	UC	Ø
Skinner, 2019[15]	N/A	Y	Y	N/A	Y	Y	Y	Y	Y	Y	Y	Y	Y	Y	+

N/A, not applicable, Y, yes, N, no, UC, unclear

*Overall quality ratings

MINUS/NEGATIVE (-) If most (six or more) of the answers to the above validity questions are “No,” the report is with a minus (-) symbol on the Evidence Worksheet. NEUTRAL (Ø) If the answers to validity criteria questions 2, 3, 6, and 7 do not indicate that the study is exceptionally strong, the report is designated with a neutral (Ø) symbol on the Evidence Worksheet.
PLUS/POSITIVE (+) If most of the answers to the above validity questions are “Yes” (including criteria 2, 3, 6, 7 and at least one additional “Yes”), the report is designated with a plus symbol (+) on the Evidence Worksheet.

Table S3. Dietary Outcomes of Included Studies

Author, year, country	Dietary outcomes
Ayaz,[1] 2018, Turkey	*Energy:* Mean EI (kcal/day): Females - FA 2377.2, NFA 2071.1; p=0.006). Males - FA 2499.7, NFA 2587.8; p=0.719.<br><br>*Macronutrients:* Protein (g/day): Females - FA 82.1, NFA 73.4; p=0.009. Males - FA 93.4, NFA 95.6; p=0.313. Total fat (g/day): Females - FA 102.9, NFA 87.0; p<0.001. Males - FA 102.6, NFA 109.1; p=0.838. SFA (g/day): Females - FA 29.5, NFA 25.6; p=0.002. Males - FA 32.2, NFA 32.9; p=0.833. MUFA (g/day): Females - FA 29.5, NFA 26.3; p=0.002. Males - FA 33.0, NFA 33.5; p=0.890. PUFA (g/day): Females - FA 35.3, NFA 29.3; p=<0.001. Males - FA 37.6, NFA 35.3; p=0.993. Cholesterol (mg/day): Females - FA 278.6, NFA 213.7; p=0.001. Males - FA 333.7, NFA 284.7; p=0.071. CHO (g/day): Females - FA 243.6, NFA 235.6; p=0.170. Males - FA 293.8, NFA 295.1; p=0.548. Sucrose (g/day): female FA 41.7, NFA 37.7; p=0.242. Males - FA 34.6, NFA 44.9; p=0.067. Fibre (g/day): Females - FA 29.8, NFA 25.5; p=0.016. Males - FA 29.8, NFA 29.6; p=0.662.<br><br>*Micronutrients:* Vitamin A (µg/day): Females - FA 2090.1, NFA 1893.5; p=0.046. Males - FA 1989.4, NFA 1968.7; p=0.892. Vitamin B1 (mg/day): Females - FA 1.28, NFA 1.12; p=0.059. Males - FA 1.32, NFA 1.34; p=0.711. Vitamin B2 (mg/day): Females - FA 1.81, NFA 1.60; p=0.100. Males - FA 1.90, NFA 1.96; p=0.753. Vitamin B12 (µg/day): Females - FA 4.4, NFA 3.6; p=0.006. Males - FA 6.2, NFA 5.4; p=0.312. Vitamin C (mg/day): Females - FA 117.1, NFA 110.5; p=0.312. Males - FA 113.0, NFA 107.5; p=0.657. Vitamin E (mg/day): Females - FA 29.5, NFA 25.9; p=0.003. Males - FA 28.1, NFA 28.3; p=0.903. Folic acid (µg/day): Females - FA 372.7, NFA 333.0; p=0.067. Males - FA 423.7, NFA 397.8; p=0.703. K (mg/day): Females - FA 3027.2, NFA 2702.4; p=0.082. Males - FA 3085.8, NFA 3027.6; p=0.570. Ca (mg/day): Females - FA 729.7, NFA 727.0; p=0.475. Males - FA 800.6, NFA 848.8; p=0.580. Mg (mg/day): Females - FA 381.6, NFA 339.9; p=0.048. Males - FA 385.1, NFA 399.8; p=0.769. Fe (mg/day): Females - FA 17.4, NFA 15.2; p=0.009. Males - FA 18.1, NFA 17.5; p=0.549. Zn (mg/day): Females - FA 11.6, NFA 10.0; p=0.008. Males - FA 12.6, NFA 12.9; p=0.335.
Ben Porat,[2] 2020, Israel	*Energy:* Mean EI (kcal/d): Pre-surgery^ - FA 3342.6 ± 100.4, NFA 2972.3 ± 42.1; p=0.053. 3-month post-surgery^ - FA 926.9 ± 104.9, NFA 869.8 ± 42.7; p=0.319. 6-month post-surgery^ - FA 911.3 ± 113.4, NFA 951.6 ± 44.2; p=0.339. 12-month post-surgery^ - FA 1073.4 ± 102.6, NFA 1129.3 ± 42.1; p=0.440.<br><br>*Macronutrients:* Protein (g/day) Pre-surgery^ - FA 132.5 ± 5.7, NFA 121.4 ± 3.3; p=0.265. 3-month post-surgery^ – FA 50.3 ± 6.0, NFA 50.1 ± 3.4; p=0.946. 6-month post-surgery^ - FA 49.5 ± 6.5, NFA 52.7 ± 3.5; p=0.511. 12-month post-surgery^ - FA 50.6

	± 6.8, NFA 57.3 ± 3.3; p=0.095. CHO (g/day): Pre-surgery^ - FA 390.9 ± 14.6, NFA 382.2 ± 7.9; p=0.748. 3-month post-surgery^ – FA 105.2 ± 15.3, NFA 93.5 ± 8.1; p=0.249. 6-month post-surgery^ - FA 104.6 ± 16.5, NFA 99.5 ± 8.4; p=0.508. 12-month post-surgery^ - FA 120.5 ± 14.9, NFA 121.6 ± 7.9; p=0.918.<br><br>^According to baseline FA diagnosis (FA, n=22; NFA, n=32)<br><br>12-month post-surgery (FA, n=12; NFA, n=29):<br><br>*Energy:*<br>EI (kcal/day): FA 1203.0 ± 339.3, NFA 1077.2 ± 215.6; p=0.161.<br><br>*Macronutrients:*<br>Protein (g/day) FA 52.3 ± 15.0, NFA 54.9 ± 15.8, p=0.618. Fat (g/day) FA 46.6 ± 19.3, NFA 44.9 ± 15.1; p=0.771. CHO (g/day): FA 148.2 ± 51.3, NFA 110.2 ± 38.3; p=0.013.
Burrows,[3]<br>2017,<br>Australia	*Foods:*<br>Core food intake for total sample: Fruit - 47% consumed >2 servings/day of fruit, 53% consumed <1 serving/day. Vegetables - 13.4% consumed >5 servings of vegetables per day. Breakfast - 70% had breakfast everyday with 27% consuming breakfast cereal daily, and 38.3% consuming breakfast cereal 'never' or 'rarely'. Milk - Two most popular milks consumed were regular full cream (38%) and low/reduced fat milk (29.9%). Non-core food intake for total sample: Take away food – 76% consumed never or rarely, 19.2% consumed 1–2 times/week and 1.3% 5–6 times/ week. Hot chips - 43% consumed 1–2 times/week. Confectionary - 51% consumed three or more times/week. Soft drink - 82.6% consumed less than one cup/week. Fruit juice - 80% consumed less than one cup/week.<br><br>Predictors of severe FA (n=168) relative to NFA (reference group): vegetable consumption OR 0.81 (95% CI 0.71, 0.93); p=0.003; soft drink consumption OR 1.36 (95% CI 1.07, 1.32); p=0.011; confectionaries 1-2 times/week OR 1.49 (95% CI 0.72, 3.09); p=0.279; confectionaries 3-4 times/week OR 1.18 (95% CI 0.54, 2.61); p=0.673; confectionaries 5-6 times/week OR 2.4 (95% CI 1.1, 5.8); p=0.034; confectionaries once/day OR 2.43 (95% CI 1.1, 5.5); p=0.033; confectionaries ≥2 times/day OR 7.1 (95% CI 3.1, 16.1); p=0.000.
Filgueras,[4]<br>2019,<br>Brazil	*Energy*:*<br>Mean EI (kJ/day) FA 8896 (95% CI 8266, 9526), NFA 8438 (95% CI 7978, 8897); p=0.021<br><br>*Macronutrients*:*<br>Protein (g/day): FA 80.6 (95% CI 74, 86), NFA 79.92 (95% CI 75, 84); p=0.042. Vegetable protein (g/day): FA 30 (95% CI 28, 32), NFA 29 (95% CI 28, 31); p=0.033. Animal protein (g/day): FA 49 (95% CI 45, 54), NFA 50 (95% CI 46, 54); p=0.05. Total fat (g/day): FA 79 (95% CI 73, 86), NFA 75 (95% CI 70, 80); p=0.029. Trans fat (g/day) 2.0 (95% CI 1.81, 2.28), NFA 1.9 (95% CI 1.77, 2.09); p=0.025. CHO (g/day): FA 274 (95% CI 254, 294), NFA 257 (95% CI 244, 270); p=0.013. Total sugar (g/day): FA 106 (95% CI 93, 119), NFA 95 (95% CI 88, 101); p=0.008. Added sugars (g/day): FA 69 (95% CI 58, 79), NFA 55 (95% CI 50, 59); p=0.004. Fructose (g/day): FA 20 (95% CI 16, 23), NFA 18 (95% CI 16,

	19); p=0.017. Fibre (g/day): FA 17.7 (95% CI 16.23, 19.04), NFA 17.8 (95% CI 16.98, 18.65); p=0.046. Na (g/day): FA 3.3 (95% CI 3.03, 3.58), NFA 3.2 (95% CI 3.06, 3.42); p=0.038.<br><br>*Energy and nutrient intakes from* unprocessed/*minimally processed foods*:*<br>Total energy (kJ/day): FA 3482 (95% CI 3242, 3723), NFA 3684 (95% CI 3500, 3862); p=0.275. Total protein (g/day): FA 43 (95% CI 40, 47), NFA 47 (95% CI 43, 51); p=0.254. Vegetable protein (g/day): FA 11 (95% CI 10, 12), NFA 11 (95% CI 11, 12); p=0.268. Animal protein (g/day): FA 32 (95% CI 29, 35), NFA 35 (95% CI 31, 39); p=0.339. Total fat (g/day): FA 21 (95% CI 19, 24), NFA 22 (95% CI 20, 24); p=0.729. CHO (g/day): FA 98 (95% CI 90, 106), NFA 103 (95% CI 98, 108); p=0.287. Total sugar (g/day): FA 25 (95% CI 21, 29), NFA 28 (95% CI 25, 31); p=0.290. Fructose (g/day): FA 0.12 (95% CI 0.16, 0.20), NFA 0.2 (95% CI 0.18, 0.21); p=0.404. Fibre (g/day): FA 7.5 (95% CI 6.46, 8.52), NFA 8.2 (95% CI 7.59, 8.74); p=0.241. Na (g/day): FA 0.9 (95% CI 0.87, 1.02), NFA 0.9 (95% CI 0.93, 1.00); p=0.638.<br><br>*Energy and nutrient intakes* from *ultra-processed food*:*<br>Total energy (kJ/day): FA 3441 (95% CI 3018, 3865), NFA 2905 (95% CI 2665, 3146); p=0.023. Total protein (g/day): FA 13 (95% CI 11, 16), NFA 10 (95% CI 9, 11); p=0.009. Vegetable protein (g/day): FA 4.6 (95% CI 3.62, 5.62), NFA 3.9 (95% CI 3.37, 4.55); p=0.041. Animal protein (g/day): FA 8.8 (95% CI 6.69, 10.93), NFA 6.3 (95% CI 5.58, 7.05); p=0.005. Total fat (g/day): FA 28 (95% CI 23, 32), NFA 23 (95% CI 20, 26); p=0.036. Trans fat (g/day): FA 0.6 (95% CI 0.03, 0.15), NFA 0.1 (95% CI 0.02, 0.19); p=0.050. CHO (g/day): FA 112 (95% CI 96, 127), NFA 93 (95% CI 85, 101); p=0.018. Total sugar (g/day): FA 63 (95% CI 52, 74), NFA 50 (95% CI 45, 55); p=0.014. Fructose (g/day): FA 11 (95% CI 8, 14), NFA 9 (95% CI 8, 10); p=0.032. Fibre (g/day): FA 1.4 (95% CI 0.97, 1.89), NFA 1.2 (95% CI 0.94, 1.49); p=0.045. Na (g/day): FA 1.1 (95% CI 0.94, 1.24), NFA 0.9 (95% CI 0.86, 1.01); p=0.027.<br><br>*Energy from ultra*-processed *food items (kJ)*:*<br>Soft drinks (kJ/day): FA 262 (95% CI 167, 357), NFA 178 (95% CI 145, 211); p=0.011. Sweetened Juices (kJ/day): FA 118 (95% CI 87, 149), NFA 116 (95% CI 91, 141); p=0.044. Sauces and condiments (kJ/day): FA 189 (95% CI 148, 229), NFA 165 (95% CI 144, 186); p=0.022. Sweetened milk drinks (kJ/day): FA 216 (95% CI 168, 265), NFA 212 (95% CI 177, 247); p=0.050. Desserts (kJ/day): FA 335 (95% CI 251, 419), NFA 295 (95% CI 250, 340); p=0.028. Cookies and savory biscuits (kJ/day): FA 996 (95% CI 730–1263), NFA 688 (95% CI 599–778); p=0.006. Sausages (kJ/day): FA 445 (95% CI 357–143), NFA 385 (95% CI 349–421); p=0.017. Corn Chips (kJ/day): FA 121 (95% CI 60–183), NFA 162 (95% CI 116–207); p=0.039. Instant noodles (kJ/day): FA 307 (95% CI 242–371), NFA 270 (95% CI 209–331); p=0.033.<br><br>AORs** (95% CI) for the association between FA and ultra-processed foods: Cookies/Biscuits OR=4.19 (1.32, 13.26), p=0.015; Sausages OR=11.77 (1.29, 107.42), p=0.029; and soft drinks OR=1.26 (0.83, 1.92), p=0.05.<br><br>*Adjusted for age and sex; critical p-values defined for multiplicity<br>**Adjusted for sugar, sodium and fat ingestion; critical p-values defined for multiplicity
Grammatikopolou,[5]<br>2018,<br>Greece	*Energy:*<br>Mean EI (kcal/kg): FA 26.7 ± 9.5, NFA 22.8 ± 5.7; p=0.284. No significant association between mYFAS score and EI (β = 0.001, 95%CI = − 0.052 to 0.054; p=0.974).

	Macronutrients:<br>Protein (g/day): FA 65.4 ± 18.1, NFA 75.2 ± 20.2; p=0.196. Fat (g/day): FA 63.9 ± 17.5, NFA 70.3 ± 16.5; p=0.268. Trans fat (g/day): FA 2.2 ± 3.0, NFA 2.2 ± 4.0; p=0.910. SFA (g/day): FA 24.2 ± 10.2, NFA 22.8 ± 7.8; p=0.926. MUFA (g/day): FA 23.0 ± 5.9, NFA 29.4 ± 12.1; p=0.117. PUFA (g/day): FA 8.5 ± 3.9, NFA 9.1 ± 3.3; p=0.418. Fibre (g/day): FA 14.3 ± 8.0, NFA 19.8 ± 41.8; p=0.233.<br><br>*Micronutrients:*<br>Na (mg/day): FA 2260.3 ± 1126.5, NFA 2819.0 ± 1032.6; p=0.115.
Keser,[6]<br>2015,<br>Turkey	*Foods:*<br><br>The ‘most addictive foods’ reported by participants (%) were chocolate (70%), carbonated beverages (59%), ice cream (58%), French fries (57%), white bread (55%), rice (53%), candy (50%), chips (48%) and pasta (43%). FA (%) reported daily consumption of chocolate (32.4%), carbonated beverages (36.6%), French fries (46.8%), white bread (95.8%), rice/pasta (46.5%), candy (43.7%), chips (38.2%); and consumption of ice cream 3-5 times a week by 47.4%.<br><br>Foods associated with FA: French fries consumption ≥ 1-2 times/week OR 2.29 (95% CI 0.81, 6.50), p=0.007; hamburger consumption ≥ 1-2 times/week OR 1.53 (95% CI 0.56, 4.21), p=0.106.
Kucukerdonmez,[7]<br>2017,<br>Turkey	*Energy:*<br>Mean EI (kcal/day): FA 2535.2 ± 887.90, NFA 1994.6 ± 700.96; p=0.001<br><br>*Macronutrients:*<br>CHO (g/day): FA 322.2 ± 142.27, NFA 238.8 ± 97.71; p=0.001. CHO (%E/day): FA 51.3 ± 7.62, NFA 48.9 ± 9.42; p=0.182. Fibre (g/day): FA 28.8 ± 16.76, NFA 22.11 ± 11.71; p=0.028. Protein (g/day): FA 86.0 ± 31.38, NFA 73.5 ± 36.55; p=0.065. Protein (%E/day): FA 14.11 ± 3.79, NFA 15.1 ± 4.48; p=0.230. Fat (g/day): FA 95.5 ± 30.61, NFA 79.9 ± 33.97; p=0.017. Fat (%E/day): FA 34.6 ± 6.47, NFA 36.0 ± 8.08; p=0.327. SFA (g/day): FA 25.7 ± 9.71, NFA 22.1 ± 10.65; p=0.082. MUFA (g/day): FA 28.9 ± 10.47, NFA 25.5 ± 11.76; p=0.128. PUFA (g/day): FA 34.4 ± 15.20, NFA 27.0 ± 14.23; p=0.016.
Lemeshow,[8]<br>2017,<br>USA	*Energy:*<br>Mean EI (kcal/day): NHS FA 1773.1 ± 579.9, NFA 1648.7 ± 532.3; NHSII FA 1882.3 ± 596.5, NFA 1802.8 ± 554.1 (significance not tested).<br><br>*Foods:*<br>AORs* (95% CI) for the association between FA and positively reinforcing foods and beverages consumed 5+ times/week compared with <1 time/month:<br>Bacon OR 1.56 (120, 2.03), p=0.001; Hamburgers OR (4.08 (2.66, 6,25), p<0.001; Hamburgers (lean) OR 1.62 (1.07, 2.44), p<0.001; Beef as a main dish OR1.54 (1.16, 2.06), p=0.001; Crackers OR 1.16 (1.06, 1.27), p=0.04; French fries

	OR 2.37 (1.59, 3.51); p<0.001; Popcorn OR 1.45 (1.11, 1.89), p<0.001; Potato/corn chips OR 1.38 (1.19–1.61), p<0.001; Pretzels OR 1.13 (1.00–1.28), p=0.005; Cake OR 1.12 (0.69, 1.82)<br><br>0.001; Candy bars OR 1.95 (1.50, 2.54), p<0.001; Candy without chocolate OR 1.28 (1.10, 1.49), p<0.001; Dark chocolate OR 0.77 (0.67, 0.89), p<0.001; Milk chocolate OR 1.69 (1.49, 1.91), p<0.001; Cookies (homemade) OR 0.75 (0.63, 0.90), p<0.001; Cookies (store) OR 1.00 (0.89, 1.13), p=0.06; Doughnuts OR 1.49 (0.95, 2.34),p<0.001; Ice cream OR 0.94 (0.78, 1.13), p=0.26; Pie OR 1.94 (0.94, 4.01), p=0.20; Sweet roll/coffee cake (homemade) OR 0.94 (0.43, 2.05), p<0.001; Sweet roll/coffee cake (store) OR 1.11 (0.72, 1.71), p<0.001; Pasta OR 0.88 (0.72, 1.08), p<0.001; White bread OR 1.13 (1.03, 1.23), p=0.02; White potato OR 0.91 (0.77, 1.06), p<0.001; White rice OR 0.46 (0.31, 0.69), p<0.001; Butter OR 1.13 (1.05, 1.22), p=0.004; Cheese (full fat) OR 0.90 (0.85, 0.96), p=0.001; Pizza OR 2.49 (1.67, 3.69), p<0.001; Sugar-Sweetened Beverages OR 0.56 (0.52, 0.61), p<0.001; Low-Calorie Beverages OR 2.38 (2.24, 2.54), p<0.001.<br><br>AORs* (95% CI) for the association between FA and Low/No Fat and/or No Sugar Foods and Beverages consumed 5+ times/week compared with <1 time/month: Popcorn (fat free) OR 2.04 (1.73, 2.40), p<0.001; Cookies (no/low fat) OR 1.08 (0.89, 1.32), p<0.001; Sherbet/frozen yogurt OR 1.16 (1.00, 1.35), p<0.001; Sweet roll/coffee cake (no/low fat) OR 2.16 (1.22, 3.85), p<0.001 artificial sweetener (excluding Splenda) OR 1.71 (1.60, 1.83), p <0.001; Splenda OR 1.76 (1.65, 1.87), p <0.001; caffeinated low calorie beverages OR 2.21 (2.07–2.35), p <0.001; decaffeinated low calorie beverages OR 2.00 (1.86–2.14), p <0.001; water OR 0.87 (0.79–0.97), p=0.002.<br><br>Consumption of apples, grapes, avocado, broccoli, corn, and peas had significant inverse associations with food addiction, AORs ranged from 0.57 0.94.<br><br>*Adjusted for age, total EI, alcohol intake, current smoking status, and depression).
Moghaddam,[9]<br>2019,<br>Iran	*Energy:*<br>Mean EI (kcal/day): FA 3393 ± 101, NFA 2754 ± 110; p<0.001.<br><br>*Macronutrients:*<br>Protein (%E/day): 116.5 ± 37, NFA 97.8 ± 39; p<0.001. CHO (%E/day): FA 483.3 ± 15, NFA 398.7 ± 16; p<0.001. Sucrose (g/day): FA 48.1 ± 29, NFA 40.5 ± 27; p=0.1. Fat (%E/day): FA 121.5 ± 45, NFA 95.4 ± 45; p<0.001. SFA (mg/day): FA 32.8 ± 14, NFA 26.7 ± 12; p<0.001. MUFA (mg/day): FA 40.5 ± 18, NFA 32.2 ± 16; p<0.001. PUFA (mg/day): FA 29.5 ± 14, NFA 22.7 ± 13; p<0.001. Trans fat (mg/day): FA 0.2 ± 0.51, NFA 0.05 ± 0.25; p=0.1. Cholesterol (mg/day): FA 320.2 ± 16, NFA 256.8 ± 116; p<0.001.
Pedram,[10]<br>2013,<br>Canada	*Macronutrients:*<br>Protein (%E/day): 19.0 ± 3.8, NFA 17.9 ± 39; p=0.04. Protein (g/kg BW): 1.2 ± 0.5, NFA 1.2 ± 0.9; p=0.89. CHO (%E/day): FA 52.2 ± 7.4, NFA 54.3 ± 8.5; p=0.07. CHO (g/kg BW): FA 3.5 ± 1.8, NFA 3.9 ± 2.7; p=0.23. Fat (%E/day): FA 26.6 ± 7.5, NFA 24.3 ± 7.2; p=0.04. Fat (g/kg BW): FA 0.8 ± 0.4, NFA 0.8 ± 0.7; p=0.59.

Pedram,[11] 2015, Canada	*Energy:*<br>Mean EI (calories/day): FA 2077.4 ± 687.6, NFA 1714.0 ± 612.0; p=0.7. EI: (calories/kg BW) FA 24.4 ± 10.9, NFA 19.5 ± 6.6; p=0.02.<br><br>*Macronutrients:*<br>Protein (%E/day): FA 19.3 ± 3.9, NFA 18.2 ± 2.6; p=0.2. Protein (g/kg BW): FA 1.1 ± 0.4, NFA 0.9 ± 0.3; p=0.2. Fat (%E/day): FA 27.1 ± 7.5, NFA 23.4 ± 4; p=0.005. Fat (g/kg BW): FA 0.7 ± 0.4, NFA 0.5 ± 0.2; p=0.004. SFA (g/kg BW): FA 0.3 ± 0.1, NFA 0.2 ± 0.1; p=0.01. Trans fat (mg/kg BW): FA 1.0 ± 0.0, NFA 0.1 ± 0.0; p=0.01. MUFA (g/kg BW): FA 0.3 ± 0.1, NFA 0.2 ± 0.1; p=0.01. PUFA (g/kg BW): FA 0.1 ± 0.1, NFA 0.1 ± 0.0; p<0.01. Omega 3 (mg/kg BW): FA 7.0 ± 0.0, NFA 5.0 ± 0.0; p=0.01. Omega 6 (mg/kg BW): FA 0.1 ± 0.0, NFA 0.03 ± 0.0; p<0.01. CHO (%E/day): FA 51.2 ± 7.1, NFA 56.3 ± 5.2; p=0.3. CHO (g/kg BW): FA 3.2 ± 1.6, NFA 2.8 ± 1; p=0.03. Sugar (g/kg BW): FA 1.4 ± 0.8, NFA 0.2 ± 0.5; p=0.03.<br><br>*Micronutrients:*<br>B1 (mg/kg BW): FA 0.02 ± 0.01, NFA 0.02 ± 0.0; p=0.04. Vit D (IU/kg BW): FA 2.5 ± 2.1, NFA 1.9 ± 1.0; p=0.04. Dihydrophylloquinone (µg/kg BW) FA 0.3 ± 0.0, NFA 0.2 ± 0.0; p=0.03. Gamma tocopherol (mg/kg BW) FA 0.3 ± 0.0, NFA 0.0 ± 0.0; p=0.04. Na (mg/kg BW) FA 26.1 ± 12.0, NFA 19.4 ± 6.3; p 0.01. Ca (mg/kg BW) FA 13.0 ± 7.1, NFA 10.0 ± 4.0; p= 0.02. P (mg/kg BW): FA 50.8 ± 21.3, NFA 41.2 ± 16.8; p=0.04. Se (mg/kg BW): FA 1.4 ± 0.6, NFA 1.1 ± 0.3; p=0.02.
Pursey,[12] 2015, Australia	*Energy:*<br>Mean EI (kcal/day): FA 1997 ± 602, NFA 2009 ± 624; AOR* 1.00 (95% CI 1.00, 1.00); p=0.59^.<br><br>*Macronutrients:*<br>Protein (%E/day): FA 19.4 ± 3.9, NFA 19.6 ± 3.6; AOR* 0.97 (0.90, 1.05); p=0.45^ Fat (%E/day): FA 35.3 ± 5.1, NFA 33.0 ± 4.5; AOR* 1.11 (1.04, 1.18); p<0.001^ SFA (%E/day): FA 14.4 ± 3.1, NFA 13.4 ± 2.4; AOR* 1.11 (0.99, 1.24); p=0.08^ PUFA (%E/day): FA 4.6 ± 1.4, NFA 4.3 ± 1.2; AOR* 1.31 (1.06, 1.61); p=0 .01^ MUFA (%E/day): FA 13.2 ± 2.3, NFA 12.3 ± 2.2; AOR* 1.22 (1.08, 1.38); p<0.001^ Cholesterol (mg/day): FA 290.5 ± 125.5, NFA 288.1 ± 124.1; AOR* 1.00 (1.00, 1.00); p=0.55^ Carbohydrate (%E/day): FA 44.1 ± 6.8, NFA 45.4 ± 6.9; AOR* 0.97 (0.93, 1.01); p=0.19^ Sugars (g/day): 112.2 ± 45.5, NFA 116.5 ± 51.4; AOR* 1.00 (0.99, 1.00); p=0.99^ Alcohol (%E/day): FA 1.5 ± 2.3, NFA 2.2 ± 2.7; AOR* 0.92 (0.81, 1.04); p=0.17^ Fibre (g/day): FA 24.9 ± 7.9, NFA 26.3 ± 8.3; AOR* 1.00 (0.97, 1.04); p=0.95^.<br><br>*Micronutrients:*<br>Vitamin A (µg/day): FA 1105.3 ± 410.1, NFA 1179.2 ± 445.6; AOR* 1.00 (1.00, 1.00); p=0.49^ Vitamin B1 (mg/day): FA 1.4 ± 0.5, NFA 1.5 ± 0.6; AOR*.64 (0.37, 1.13); p=0.12^ Vitamin B2 (mg): FA 2.0 ± 0.8, NFA 2.3 ± 0.8; AOR* 0.79 (0.55, 1.14); p=0.21^ Vitamin B3 (mg/day): FA 40.7 ± 12.8, NFA 42.2 ± 13.9; AOR* 1.00 (0.97, 1.02); p=0.77^ Vitamin C (mg/day): FA 151.5 ± 70.7, NFA 152.5 ± 61.9; AOR* 1.00 (1.00, 1.01); p=0.34^ Folate (µg/day): FA 286.7 ± 89.5, NFA

	316.4 ± 100.8; AOR* 1.00 (1.00, 1.00); p=0.33^ Na (mg/day): FA 2011.8 ± 823.5, NFA 2015.0 ± 745.7; AOR* 1.00 (1.00, 1.00); p=0.99^ K (mg/day): FA 3300.5 ± 907.3, NFA 3373.5 ± 976.7; AOR* 1.00 (1.00, 1.00); p=0.52^ Mg (mg/day): FA 375.6 ± 102.6, NFA 394.6 ± 106.1; AOR* 1.00 (1.00, 1.00); p=0.87^ Ca (mg/day): FA 1078.0 ± 430.8, NFA 1124.3 ± 406.3; AOR* 1.00 (1.00, 1.00); p=0.99^ P (mg/day): FA 1543.0 ± 485.1, NFA 1587.0 ± 499.1; AOR* 1.00 (1.00, 1.00); p=0.94^ Fe (mg/day): FA 12.2 ± 3.3, NFA 13.2 ± 4.0; AOR* 0.96 (0.88, 1.04); p=0.27^ Zn (mg/day): FA 12.2 ± 4.0, NFA 12.7 ± 4.3; AOR* 0.98 (0.91, 1.05); p=0.51^<br><br>*Foods/food groups:*<br>Core foods (%E/day): FA 62.3 ± 16.5, NFA 67.8 ± 12.9; AOR* 0.98 (95% CI 0.96, 1.00); p=0.06^ Non-core foods (%E/day): FA 37.7 ± 16.5, NFA 32.2 ± 12.9; AOR* 1.02 (95% CI 1.00, 1.04); p=0.06<br><br>Vegetables (%E/day): FA 8.2 ± 4.1, NFA 8.5 ± 3.9; AOR* 0.99 (95% CI 0.92, 1.06); p=0.71^. Fruit (%E/day): FA 8.6 ± 8.1, NFA 8.4 ± 5.6; AOR* 1.02(95% CI 0.98, 1.07); p=0.37^ Meat (%E/day): FA 15.4 ± 7.6, NFA14.7 ± 8.0; AOR* 1.00 (95% CI 0.97, 1.04); p=0.99^ Grains (%E/day): FA 15.4 ± 7.6, NFA 19.5 ± 7.6; AOR* 0.93 (95% CI 0.90. 0.97); p<0.001^ Dairy (%E/day): FA 10.0 ± 6.4, NFA 11.3 ± 6.6; AOR* 0.98 (95% CI 0.93, 1.02); p=0.27^ Sweet drink (%E/day): FA 1.9 ± 3.4, NFA 3.1 ± 5.1; AOR* 0.91 (95% CI 0.83, 1.00); p=0.05^ Savoury packaged snacks (%E/day): FA 3.2 ± 3.5, NFA 2.3 ± 2.4; AOR* 1.10 (95% CI 1.00, 1.21); p=0.06^ Candy (%E/day): FA 8.9 ± 8.2, NFA 5.8 ± 5.3; AOR* 1.06 (95% CI 1.01, 1.10); p=0.01^ Baked sweet products (%E/day): FA 5.6 ± 5.8, NFA 4.9 ± 4.0; AOR* 1.01 (95% CI 0.95, 1.08); p=0.69^ Take-out (%E/day): FA 9.8 ± 9.1, NFA 7.2 ± 5.1; AOR* 1.05 (95% CI 1.00, 1.09); p=0.03^ Breakfast cereal (%E/day): FA 4.1 ± 4.1, NFA 6.5 ± 4.9; AOR* 0.91 (95% CI 0.85, 0.97); p=0.003^<br><br>*Diet quality:*<br>ARFS (score out of 73): FA 32.6 ± 9.5, NFA 35.9 ± 9.2; AOR* 0.97 (95% CI 0.94, 1.00); p=0.05<br><br>*Odds of FA diagnosis adjusted for age, sex and BMI category. ^ Significant at p = 0.00125.
Schulte,[13]<br>2018,<br>USA	*Energy:*<br>Mean EI (kcal/day): FA 1939.19 ± 1193.20, NFA 1250.12 ± 783.44; p = 0.002, η^2 = 0.06. Positive association between YFAS and EI (r_s = 0.23, p = 0.003).<br><br>*Macronutrients:*<br>Total fat (g/day): FA 71.49 ± 46.17, NFA 47.12 ± 31.83; p=0.002, η^2 = 0.05. SFA (g/day): FA 23.31 ± 15.48, NFA 15.26 ± 10.12; p=0.003, η^2 =0.05. Trans fat (g/day): FA 6.39 ± 4.43, NFA 3.96 ± 3.38; p=0.003, η^2 =0.05. CHO (g/day): FA 264.30 ± 159.88, NFA 168.59 ± 110.34; p=0.001, η^2 = 0.06. Total sugar (g/day): FA 146.69 ± 85.18, NFA 93.67 ± 68.44; p=0.003, η^2 = 0.05. Added sugar (g/day): FA 19.04 ± 10.63, NFA 12.32 ± 10.62; p=0.001, η^2 = 0.06. Positive associations between YFAS and total fat (r_s = 0.26, p=0.001), SFA (r_s = 0.25, p=0.001), trans fat (r_s = 0.31, p=0.001), total CHO (r_s = 0.20, p = 0.008), total sugar (r_s = 0.16, p=0.03), and added teaspoons of sugar (r_s = 0.18, p=0.01.
Sengor,[14]<br>2020,<br>Turkey	*Energy:*<br>Mean EI (kcal/day): Females - FA 2173.0 ± 879.4, NFA 1883.0 ± 666.0; p=0.013. Males - FA 2893 ± 654.1, NFA 2640.8 ± 702.0; p=0.913.

Macronutrients:
Protein (g/day): Females - FA 74.4 ± 27.7, NFA 78.5 ± 33.4; p=0.584. Males - FA 108.4 ± 27.5, NFA 103.4 ± 35.0; p=0.279. Total fat (g/day): Females - FA 94.0 ± 40.5, NFA 84.7 ± 29.9; p=0.029. Males - FA 121.3 ± 32.4, NFA 112.7 ± 34.7; p=0.445. Cholesterol (mg/day): Females - FA 300.6 ± 135.5, NFA 322.4 ± 139.6; p=0.403. Males - FA 401.9 ± 149.1, NFA 395.5 ± 181.7; p=0.390. CHO (g/day): Females - FA 247.0 ± 109.0, NFA 194.5 ± 86.1; p=0.009. Males - FA 322.4 ± 92.3, NFA 288.3 ± 87.0; p=0.451. Fibre (g/day): Females - FA 24.6 ± 8.8, NFA 26.8 ± 16.2; p=0.853. Males - FA 33.9 ± 13.3, NFA 31.3 ± 13.6; p=0.219.

Significant positive weak correlations between YFAS score and EI, CHO and fat intake (r=0.228, p<0.001; r=0.222, p<0.001; r=0.225, p<0.001 respectively); in females (r=0.214, p=0.002; r=0.254, p<0.001; r=0.197, p=0.004 respectively) and in males (r=0.225, p=0.001; r=0.171, p=0.031; r=0.225, p<0.001 respectively).

Micronutrients:
Vitamin A (µg/day): Females - FA 1359.0 ± 1078.6, NFA 1435.0 ± 1310.2; p=0.801. Males - FA 3045.0 ± 2303.0, NFA 2589.0 ± 2265.4; p=0.222). Vitamin E (mg/day): Females - FA 20.0 ± 8.7, NFA 20.4 ± 8.9; p= 0.075. Males - FA 26.1 ± 9.6, NFA 24.5 ± 10.0; p=0.274. Vitamin B12 (µg/day): Females - FA 6.4 ± 4.5, NFA 6.7 ± 5.6; p=0.778. Males - FA 13.0 ± 9.7, NFA 11.9 ± 8.9; p=0.672. Vitamin B1 (mg/day): Females - FA 0.9 ± 0.3, NFA 0.8 ± 0.3; p=0.533. Males - FA 1.2 ± 0.3, NFA 1.1 ± 0.3; p=0.808. Vitamin B2 (mg/day): Females - FA 1.4 ± 0.5, NFA 1.4 ± 0.6; p=0.954. Males - FA 2.1 ± 0.7, NFA 1.9 ± 0.7; p=0.228. Vitamin B3 (mg/day): Females - FA 14.7 ± 6.0, NFA 14.8 ± 5.9; p=0.994. Males - FA 20.9 ± 7.5, NFA 20.3 ± 7.4; p=0.670. Vitamin B6 (mg/day): Females - FA 1.7 ± 0.6, NFA 1.6 ± 0.6; p=0.640. Males - FA 2.1 ± 0.6, NFA 2.1 ± 0.7; p=0.566. Vitamin C (mg/day): Females - FA 49.8 ± 34.8, NFA 61.3 ± 39.7; p=0.036. Males - FA 64.0 ± 39.7, NFA 63.8 ± 36.8; p=0.790. Folic acid (µg/day): Females - FA 241.7 ± 83.4, NFA 233.8 ± 92.3; p=0.275. Males - FA 350.2 ± 76.4, NFA 312.4 ± 88.4; p=0.341. K (mg/day): Females - FA 2532.0 ± 988.7, NFA 2560.0 ± 1015.1; p=0.835. Males - FA 3230.1 ± 914.9, NFA 3120.8 ± 988.2; p=0.897. Ca (mg/day): Females - FA 619.4 ± 252.1, NFA 686.4 ± 355.3; p=0.360. Males - FA 837.0 ± 201.0, NFA 792.4 ± 292.4; p=0.233. Mg (mg/day): Females - FA 306.6 ± 109.8, NFA 296.2 ± 103.8; p=0.334. Males - FA 388.2 ± 109.9, NFA 366.1 ± 114.6; p=0.709. P (mg/day): Females - FA 1226.0 ± 413.8, NFA 1232.0 ± 435.8; p=0.881. Males - FA 1659.6 ± 349.3, NFA 1558.3 ± 458.4; p=0.087. Fe (mg/day): Females - FA 11.9 ± 4.1, NFA 11.4 ± 4.3; p=0.821. Males - FA 16.1 ± 3.8, NFA 14.9 ± 4.1; p=0.723. Zn (mg/day): Females - FA 10.3 ± 3.6, NFA 9.9 ± 3.4; p=0.760. Males - FA 14.6 ± 3.3, NFA 13.6 ± 3.8; p=0.205.

Food groups:
Milk and dairy products (g/day): Females - FA 270.8 ± 169.6, NFA 226.3 ± 111.5; p=0.122. Males - FA 243.5 ± 116.4, NFA 304.6 ± 207.0, p=0.259. Bread and grains; (g/day): Females - FA 306.0 ± 128.5, NFA 296.7 ± 130.2; p=0.924. Males - FA 329.0 ± 147.5, NFA 295.1 ± 135.2; p=0.565. Oily seeds (g/day): Females - FA 42.8 ± 25.0, NFA 42.2 ± 32.7; p=0.565. Males - FA 71.5 ± 50.2, NFA 37.8 ± 30.6; p<0.001. Meat and egg (g/day): Females - FA 125.1 ± 58.9, NFA 102.7 ± 70.6; p=0.002. Males - FA 154.6 ± 89.2, NFA 175.6 ± 110.4; p=0.508. Sausage (g/day): Females - FA 8.9 ± 7.4, NFA 8.2 ± 8.2; p=0.018. Males - FA 11.0 ± 10.9, NFA 8.8 ± 8.9; p=0.332. Vegetables (g/day): Females - FA 96.5 ± 71.1, NFA 87.6 ± 92.4; p=0.052. Males - FA 115.2 ± 110.7, NFA 162.6 ± 137.2; p=0.919. Potato, starch and mushroom (g/day): Females - FA 118.1 ± 59.2, NFA 105.0 ± 71.1; p=0.080. Males - FA 140.0 ± 47.9, NFA 127.1 ± 68.2; p=0.279.

	Fruits (g/day): Females - FA 83.7 ± 66.2, NFA 85.1 ± 69.6; p=0.974. Males - FA 95.5 ± 71.2, NFA 134.8 ± 129.2; p=0.207. Sweets (g/day): Females - FA 66.3 ± 43.0, NFA 42.5 ± 37.8; p=0.054. Males - FA 53.0 ± 46.9, NFA 78.0 ± 61.0; p=0.066. Cakes, cookies and biscuits (g/day): Females - FA 27.5 ± 20.1, NFA 18.1 ± 18.4; p=0.001. Males - FA 20.7 ± 26.3, NFA 27.0 ± 21.3; p=0.034. Oil and fat (g/day): Females - FA 31.1 ± 14.0, NFA 30.8 ± 16.3; p=0.773. Males - FA 40.1 ± 26.2, NFA 28.5 ± 16.5; p=0.041.
Skinner,[15] 2019, Australia	*Food groups:* Core foods (%E/day): FA 51.5 ± 21.75, NFA 70.25 ± 11.05; p=0.026. Non-core foods (%E/day): FA 48.5 ± 21.75, NFA 29.75 ± 11.05; p=0.026. *Diet quality:* ARFS (score out of 73): FA 33.33 ± 6.92, NFA 39.0 ± 6.51; p=0.107.

AOR, Adjusted Odds Ratio; ARFS, Australian Recommended Food Score, BMI, Body Mass Index; BW, body weight; Ca, calcium; CHO, carbohydrate; CI, confidence interval, E, energy; EI, energy intake; FA, food addicted; Fe, iron; FFQ, Food Frequency Questionnaire; IQR, Interquartile Range; K, potassium; Mg, magnesium; MUFA, monounsaturated fatty acids; mYFAS, modified Yale Food Addiction Scale; Na, sodium; N/A, not applicable; NFA, non-food addicted; NR, not reported; OR, Odds Ratio; P, potassium; PUFA, polyunsaturated fatty acids; RCT, randomised controlled trial; SD, standard deviation; Se, selenium; SFA, saturated fatty acids; YFAS, Yale Food Addiction Scale; YFAS-C, Yale Food Addiction Scale for Children, Zn, zinc.

[a]Core foods include fruit vegetables, dairy, meat/protein and carbohydrate/grain-based foods. [b]Non-core foods include discretionary choice foods with added or high amounts of salt, sugar and/or fat.

References

1. Ayaz A, Nergiz-Unal R, Dedebayraktar D, Akyol A, Pekcan AG, Besler HT, et al. How does food addiction influence dietary intake profile? PloS one. 2018;13(4):e0195541. 10.1371/journal.pone.0195541
2. Ben-Porat T, Weiss R, Sherf-Dagan S, Rottenstreich A, Kaluti D, Khalaileh A, et al. Food Addiction and Binge Eating During One Year Following Sleeve Gastrectomy: Prevalence and Implications for Postoperative Outcomes. Obesity surgery. 2020. http://dx.doi.org/10.1007/s11695-020-05010-4
3. Burrows T, Hides L, Brown R, Dayas CV, Kay-Lambkin F. Differences in Dietary Preferences, Personality and Mental Health in Australian Adults with and without Food Addiction. Nutrients. 2017;9(3). 10.3390/nu9030285
4. Filgueiras AR, de Almeida VBP, Nogueira PCK, Domene SMA, da Silva CE, Sesso R, et al. Exploring the consumption of ultra-processed foods and its association with food addiction in overweight children. Appetite. 2019;135:137-45. http://dx.doi.org/10.1016/j.appet.2018.11.005
5. Grammatikopoulou MG, Gkiouras K, Markaki A, Theodoridis X, Tsakiri V, Mavridis P, et al. Food addiction, orthorexia, and food-related stress among dietetics students. Eating and Weight Disorders. 2018;23(4):459-67. 10.1007/s40519-018-0514-1
6. Keser A, Yuksel A, Yesiltepe-Mutlu G, Bayhan A, Ozsu E, Hatun S. A new insight into food addiction in childhood obesity. Turkish Journal of Pediatrics. 2015;57(3):219-24.
7. Kucukerdonmez O, Urhan M, Altin M, Haciraifoglu O, Yildiz B. Assessment of the relationship between food addiction and nutritional status in schizophrenic patients. Nutritional Neuroscience. 2019;22(6):392-400. https://dx.doi.org/10.1080/1028415X.2017.1392429
8. Lemeshow AR, Rimm EB, Hasin DS, Gearhardt AN, Flint AJ, Field AE, et al. Food and beverage consumption and food addiction among women in the Nurses' Health Studies. Appetite. 2018;121:186-97. 10.1016/j.appet.2017.10.038
9. Moghaddam SAP, Amiri P, Saidpour A, Hosseinzadeh N, Abolhasani M, Ghorbani A. The prevalence of food addiction and its associations with plasma oxytocin level and anthropometric and dietary measurements in Iranian women with obesity. Peptides. 2019;122:170151. 10.1016/j.peptides.2019.170151
10. Pedram P, Wadden D, Amini P, Gulliver W, Randell E, Cahill F, et al. Food addiction: its prevalence and significant association with obesity in the general population. PloS one. 2013;8(9):e74832. 10.1371/journal.pone.0074832
11. Pedram P, Sun G. Hormonal and dietary characteristics in obese human subjects with and without food addiction. Nutrients. 2015;7(1):223-38. 10.3390/nu7010223
12. Pursey KM, Collins CE, Stanwell P, Burrows TL. Foods and dietary profiles associated with 'food addiction' in young adults. Addictive Behaviors Reports. 2015;2:41-8. http://dx.doi.org/10.1016/j.abrep.2015.05.007
13. Schulte EM, Jacques-Tiura AJ, Gearhardt AN, Naar S. Food addiction prevalence and concurrent validity in African American adolescents with obesity. Psychology of Addictive Behaviors. 2018;32(2):187-96. 10.1037/adb0000325
14. Sengor G, Gezer C. The association between food addiction, disordered eating behaviors and food intake. Revista De Nutricao-Brazilian Journal of Nutrition. 2020;33:10. 10.1590/1678-9865202033e190039
15. Skinner JA, Garg ML, Dayas CV, Burrows TL. Is weight status associated with peripheral levels of oxytocin? A pilot study in healthy women. Physiology and Behavior. 2019;212:112684. 10.1016/j.physbeh.2019.112684
